# Synthesis, Characterization, and Comparative Study on Norbornene Polymerization of CNN and PCN Pincer Palladium Complexes

**DOI:** 10.3390/molecules30071530

**Published:** 2025-03-30

**Authors:** Huizhu Wang, Jin-Kui Liu, Yi-Dong Wang, Xin-Qi Hao, Mao-Ping Song, Jun-Fang Gong, Hui Jiang

**Affiliations:** Pingyuan Laboratory, College of Chemistry, Zhengzhou University, Zhengzhou 450001, China; 15238011312@163.com (H.W.); liujinkui28@163.com (J.-K.L.); 15122938537@163.com (Y.-D.W.); xqhao@zzu.edu.cn (X.-Q.H.); mpsong@zzu.edu.cn (M.-P.S.)

**Keywords:** CNN pincer, PCN pincer, palladium-catalyzed, vinyl-type polynorbornene

## Abstract

Several CNN pincer Pd(II) complexes including chiral complexes **1a**–**e** with 2-phenyl-6-(oxazolinyl)pyridines and achiral ones **2a**–**c** with *N*-substituted-2-aminomethyl-6-phenylpyridines were prepared. In addition, the preparation of the achiral PCN pincer Pd(II) complexes **3a**–**e** with aryl-based phosphinite–imine ligands and chiral **4a**–**c** with aryl-based phosphinite–imidazoline ligands was also performed. Among them, the PCN Pd(II) pincers **3a**–**e** were new complexes and were readily synthesized from commercially available materials in only two steps. The new complexes were characterized through elemental analyses, namely ^1^H NMR, ^13^C{^1^H} NMR, and ^31^P{^1^H} NMR spectroscopies. Furthermore, the molecular structure of complex **3a** was determined via X-ray single-crystal diffraction analysis. In the presence of EtAlCl_2_, Et_2_AlCl, or methylaluminoxane (MAO), the CNN pincer Pd(II) complexes and PCN pincer Pd(II) complexes exhibited excellent activities and monomer conversion rates in norbornene addition polymerization. Surprisingly, the CNN pincer Pd(II) complexes exhibited a higher conversion rate (99.5%) with Et_2_AlCl as the cocatalyst, while the PCN pincer Pd(II) complexes showed a higher conversion rate (98.8%) with MAO.

## 1. Introduction

**Chart 1:**
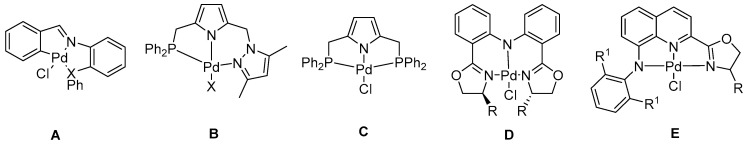
Representative pincer palladium(II) catalysts for norbornene polymerization reported in the literature: (**A**) CNX pincer Pd(II) catalysts, (**B**) PNN pincer Pd(II) catalysts, (**C**) PNP pincer Pd(II) catalyst, (**D**,**E**) NNN pincer Pd(II) catalysts.

Polynorbornene (PNB) has been widely used in production and life since PNB shows numerous excellent properties, for instance beneficial UV resistance, outstanding transparency, high glass transition temperature, thermal stability, low dielectric loss, and optical birefringence [1,2,3]. The design and synthesis of highly efficient catalysts based on late-transition metals are a crucial strategy for the development of norbornene polymeric materials [4,5,6,7,8,9,10,11,12]. Late-transition metal catalysts bearing tridentate ligands have been extensively used in the field of metal-catalyzed olefin polymerization because of their better stability in air and great tolerance toward olefin polymerization [13,14,15,16,17,18,19,20]. In particular, the corresponding palladium(II) complexes are a type of outstanding catalysts and have been applied to ethylene polymerization [21], ethylene/10-undecen-1-ol copolymerization [22], norbornene (NB) polymerization [23,24,25,26,27,28,29,30,31,32,33,34], as well as the copolymerization of norbornene [35]. Among them, the palladium(II) complexes used to catalyze the polymerization of norbornene were the most reported and usually showed high activities. For example, the Jin group [25] reported that palladium(II) complexes (Chart 1, structure **A**) containing soft phosphorous or sulfur donors showed high activities with MAO for norbornene addition polymerization (up to 8.78 × 10^6^ g of PNB (mol of Pd)^−1^h^−1^). Several PNN (Chart 1, structure **B**) [26] and PNP (Chart 1, structure **C**) [27] pincer palladium(II) complexes were synthesized by the Ganesan Mani group, which showed high activities up to 10^7^ g of PNB (mol of Pd)^−1^h^−1^ in the presence of MMAO or EtAlCl_2_. Additionally, catalysts containing hard atoms (N and O) in the sidearm were investigated for their activities in NB polymerization. For instance, bis(oxazoline)-liganded NNN pincer palladium(II) (Chart 1, structure **D**) complexes have been proved to be effective for norbornene polymerization [30]. Recently, the Shi group [33] explored a family of Pd(II) complexes bearing oxazoline (Chart 1, structure **E**), which could be used as catalysts for norbornene polymerization, with catalytic activities up to 1.15 × 10^7^ g of PNB (mol of Pd)^−1^h^−1^.

**Chart 2:**
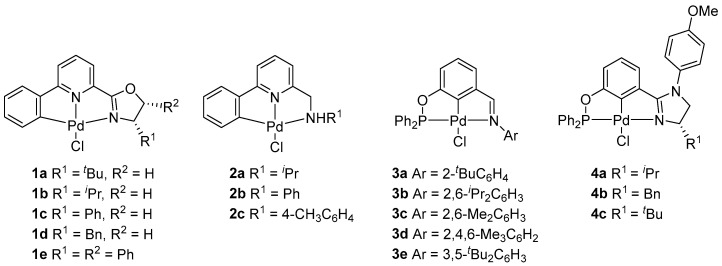
CNN and PCN pincer palladium(II) complexes for norbornene polymerization.

In recent decades, we have been working on the synthesis and applications of tridentate-coordinated pincer metal complexes [36,37,38,39,40,41,42,43,44,45]. For instance, chiral oxazoline-containing CNN Pd pincer **1** was found to be a stereoselective catalyst for the asymmetric allylation of isatins with allyltributyltin (up to 86% *ee*) and the Suzuki–Miyaura reaction (up to 68% *ee*) [36]. While the related achiral CNN pincer Pd complex **2** with *N*-substituted-2-aminomethyl-6-phenylpyridines CNN pincer Pd(II) complexes (Chart 2, **2**) exhibited good activities in the allylation of aldehydes as well as three-component allylation of aldehydes, arylamines, and allyltributyltin [37]. The achiral phosphinite–imine complexes were effective catalysts for the Suzuki–Miyaura and copper-free Sonogashira cross-coupling reactions [38]. In addition, it was proved that the chiral PCN pincer Pd complexes with aryl-based phosphinite–imidazoline ligand **4** were also applied to asymmetric Suzuki–Miyaura reaction, albeit with rather modest enantioselectivities (up to 32% *ee*) [39]. All of these Pd pincer complexes could be conveniently and readily synthesized, generally in 2–3 steps. However, the potential of these complexes in olefin polymerization has not been explored. In continuation of our research interest in pincer chemistry [36,37,38,39,40,41,42,43,44,45], herein, we synthesized a series of known and new pincer Pd(II) complexes including achiral and chiral CNN and PCN Pd(II) pincers **1**–**4** (Chart 2) and investigated their catalytic properties in NB polymerization in the presence of EtAlCl_2_, Et_2_AlCl, or MAO.

## 2. Results and Discussion

### 2.1. Synthesis and Characterization of New Achiral PCN Pincer Pd (II) Complexes 3a–e

**Scheme 1:**
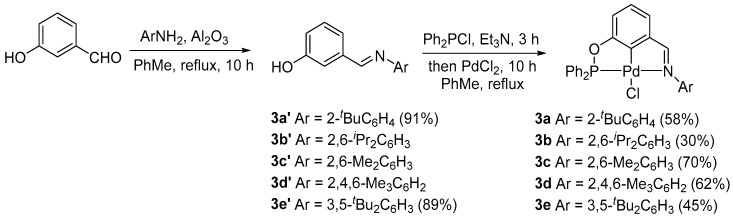
Synthesis of the achiral PCN pincer Pd(II) complexes **3a**–**e**.

**Figure 1:**
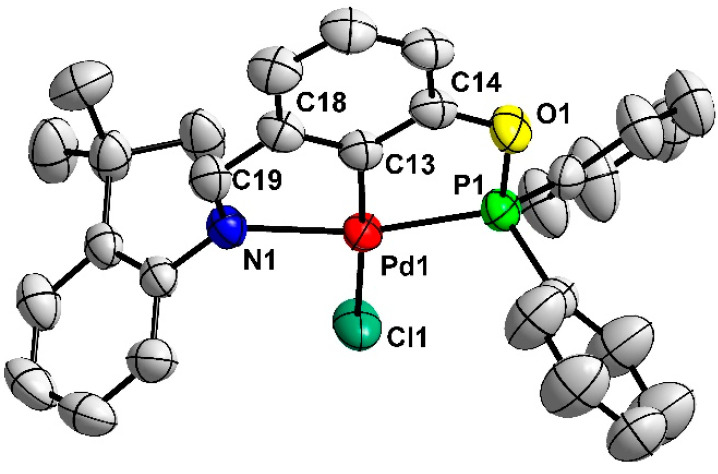
Molecular structure of complex **3a** with ellipsoids drawn at the 50% probability level. (Hydrogen atoms are omitted for clarity; one of the two independent molecules is shown.) Selected bond lengths (Å) and angles (deg) are as follows: Pd(1)-C(13) 1.951(4), Pd(1)-Cl(1) 2.3608(11), Pd(1)-N(1) 2.163(3), Pd(1)-P(1) 2.2016(11), C(13)-Pd(1)-Cl(1) 175.79(12), C(13)-Pd(1)-N(1) 79.14(15), C(13)-Pd(1)-P(1) 79.27(13), N(1)-Pd(1)-Cl(1) 98.05(9), N(1)-Pd(1)-P(1) 158.07(9), and P(1)-Pd(1)-Cl(1) 103.71(4).

The new complexes with aryl-based phosphinite–imine ligands **3a**–**e** were easily prepared when starting from commercially available 3-hydroxybenzaldehyde as they could be made in only two steps according to the method previously reported by us [38]. Firstly, 3-hydroxybenzaldehyde reacted separately with various substituted aromatic primary amines to afford the corresponding imines **3a′**–**e′** (Scheme 1). Subsequently, a one-pot phosphorylation/palladation reaction of PCN pincer preligands **3a′**–**e′** was carried out via treatment with Ph_2_PCl in the presence of the Et_3_N base in refluxing toluene for phosphorylation followed by in situ palladation with PdCl_2_, affording the complexes **3a**–**e** with 30–70% yields. The new complexes **3a**–**e** were fully characterized through ^1^H NMR, ^13^C{^1^H} NMR, and ^31^P{^1^H} NMR spectroscopies, as well as elemental analyses, which were consistent with the expected structures of these complexes. In addition, the molecular structure of complex **3a** was determined via X-ray single-crystal diffraction analysis, which unambiguously confirmed the PCN tridentate coordination mode in the complex. The molecule is shown in Figure 1. The Pd(II) center adopts a typical distorted-square-planar geometry, and the bond lengths and angles around the Pd center are comparable to those in the homologous PCN Pd pincers [38].

### 2.2. Norbornene Polymerization

#### 2.2.1. NB Polymerization Optimization with CNN Pd(II)/EtAlCl_2_

EtAlCl_2_ is a common and inexpensive cocatalyst; therefore, it was initially selected for the experiment, and the results are shown in Table 1. To understand the role of the catalysts, preliminary blank experiments were carried out with complex **1a**. The control experiment using EtAlCl_2_ without **1a** did not produce PNB (Table 1, entry 1). When the reaction was carried out with 1 µmol of pincer Pd(II) complex **1a** and 2000 equiv. of EtAlCl_2_ in 1,2-dichlorobenzene at 40 °C for 30 min, a 72.9% monomer conversion rate and a moderate activity of 1.50 × 10^6^ g of PNB (mol of Pd)^−1^h^−1^ were obtained (Table 1, entry 2). This result demonstrates that the pincer Pd(II) complex and cocatalyst synergistically catalyzed polymerization, which was consistent with the literature reports [1,12,20,22]. Elevating or reducing the Al/Pd ratio resulted in a slight decrease in the monomer conversion rates (Table 1, entries 3–4). In addition, the reaction temperature had a significant effect on the initiation and stability of the active species [31]. When the polymerization was conducted at 0 °C, the monomer conversion rate was reduced to 25.3% with an activity of 0.52 × 10^6^ g of PNB (mol of Pd)^−1^h^−1^, compared to that achieved at the optimal temperature of 20 °C (a monomer conversion rate of 80.3% and an activity of 1.65 × 10^6^ g of PNB (mol of Pd)^−1^h^−1^, Table 1, entries 5–6). Further increasing the polymerization temperature to 60 and 80 °C only slightly decreased the polymer activities (Table 1, entries 7–8), demonstrating the outstanding thermostability of the active species. The polymerization time had a significant influence on monomer conversion rates and catalytic activities. When the polymerization time was shortened from 30 min to 5 min, **1a** exhibited the highest activity of 9.22 × 10^6^ g of PNB (mol of Pd)^−1^h^−1^, and the monomer conversion rate was only 74.6% for NB polymerization (Table 1, entries 9–11). Considering the catalytic activity and monomer conversion rate, we chose 10 min as the optimized polymerization time. Then, when changing the amount of catalyst, the monomer conversion rate did not increase (Table 1, entries 12–13). Therefore, the optimum catalytic conditions were determined to be those of entry 10. With the optimal conditions, all of the other CNN pincer Pd(II) complexes **1b**–**e** and **2a**–**c** could catalyze norbornene polymerization with moderate monomer conversion rates (65.7–77.4%) and catalytic activities (4.06–4.78 × 10^6^ g of PNB (mol of Pd)^−1^h^−1^) (Table 1, entries 14–20). We found that the catalytic activities order of CNN complexes **1a**–**e** containing oxazoline coordination was **1a** > **1b** ≈ **1c** ≈ **1d** ≈ **1e**; also, the catalytic activity was almost unaffected by the steric hindrance effect of the substituents on the oxazoline ring. We suspected that this might be due to the strong rigidity of this structure, which cannot rotate in the space [46]. The order of catalytic activities of complexes **2a**–**c** was **2c** > **2b** > **2a**. It might be that the rotation of the substituent on the amine group changed the steric hindrance of the catalyst’s active center, which resulted in different polymerization activities. According to previous reports, a possible polymerization mechanism had been speculated (Scheme S1) [30,32].

#### 2.2.2. NB Polymerization Optimization with CNN Pd(II)/Et_2_AlCl

Owing to the unsatisfactory results of CNN pincer Pd(II) complexes with EtAlCl_2_ as the cocatalyst, for higher monomer conversion rates and activities, the cocatalyst Et_2_AlCl was applied to the experiment. We chose **1a** as the pre-catalyst to investigate the optimized conditions, and the results are shown in Table 2. Upon activation with 2000 to 1500 equiv. of Et_2_AlCl, a monomer conversion rate of up to 95.9% was achieved for NB polymerization (Table 2, entries 1–2). When the Al/Pd ratio was reduced from 1500 to 500, relatively low monomer conversion rates were obtained (Table 2, entries 3–4). A near-quantitative monomer conversion rate of 99.5% and a higher activity of 7.69 × 10^6^ g of PNB (mol of Pd)^−1^h^−1^ were obtained when the temperature was reduced to 20 °C (Table 2, entry 6). While elevating the temperature to 80 °C, the monomer conversion rate dramatically reduced to 5.2% (Table 2, entries 7–8). Subsequently, when the amount of catalyst was reduced to 0.8 μmol, the monomer conversion rate declined to 87.2% (Table 2, entry 9). When the reaction time was shortened to 6 min, the monomer conversion rate decreased to 93.2% (Table 2, entry 10). Therefore, the optimal conditions were determined to be an Al/Pd ratio of 1500, a temperature of 20 °C, a reaction time of 8 min, and 1 μmol of the pre-catalyst. Under the optimal conditions, the catalytic behaviors of the other CNN pincer Pd(II) complexes were further investigated. This study found that all CNN pincer Pd(II) complexes could perform efficient catalytic NB polymerization in the presence of Et_2_AlCl (Table 2, entries 12–18). Notably, the monomer conversion rates for NB polymerization catalyzed by **1b**, **1c**, and **2c** exceeded 97.3% (Table 2, entries 12–13, 18).

#### 2.2.3. NB Polymerization Optimization with PCN Pd(II)/EtAlCl_2_

Next, PCN pincer Pd(II) complexes **3** and **4** were synthesized, and their potential applications in the polymerization of norbornene were investigated. We chose EtAlCl_2_ as the cocatalyst, and the results are shown in Table 3. First, we explored the effect of the Al/Pd ratio on the catalyst activities. When the Al/Pd ratio was 1000, a monomer conversion rate of 25.7% was obtained (Table 3, entry 1). Increasing the Al/Pd ratio to 2000 resulted in a higher monomer conversion rate of 68.9% (Table 3, entries 2–3). Next, we explored the effect of the polymerization temperature on catalyst activities. When elevating or lowering the reaction temperature, the monomer conversion rates had a significant reduction (Table 3, entries 5–8). Prolonging the polymerization time to 30 min with higher monomer conversion rates resulted in lower activities (Table 3, entry 9 vs. entry 3). When the polymerization time was further increased to 60 min, the monomer conversion rate was almost unchanged (Table 3, entry 10). Finally, when changing the amount of catalyst, the monomer conversion rates did not increase (Table 3, entries 11–12). Therefore, entry 9 was confirmed as the optimum catalytic conditions. Under the optimal conditions, all other PCN pincer Pd(II) complexes exhibited moderate monomer conversion rates and catalytic activities (Table 3, entries 13–19). It had been observed that the difference in the ligand skeleton of the pre-catalyst led to significant differences in catalytic activity (Table 1 vs. Table 3). The monomer conversion rates (65.7–79.2%) and catalytic activities (4.06–4.89 × 10^6^ g of PNB (mol of Pd)^−1^h^−1^) of the polymer given by CNN pincer Pd(II) complexes **1** and **2** were greater than that of PCN pincer Pd(II) complexes **3** and **4** (monomer conversion rates: 25.9–85.3%; catalytic activities: 0.53–1.76 × 10^6^ g of PNB (mol of Pd)^−1^h^−1^, respectively). We surmised that the decrease in activities with the PCN pincer Pd(II) complexes could be explained by more steric crowding around the active metal atom. The three-dimensional orientation of the -PPh_2_ substituents in the PCN pincer Pd(II) complexes effectively shields the active metal center, thereby hindering the polymerization of the sterically hindered norbornene monomers [26].

#### 2.2.4. NB Polymerization Optimization with PCN Pd(II)/MAO

Although EtAlCl_2_ initiated NB polymerization, it resulted in only moderate monomer conversion rates and catalytic activities. The importance of the MAO in palladium-catalyzed norbornene polymerization is widely recognized [47]. Hence, we selected MAO as the cocatalyst, and the results are summarized in Table 4. The polymerization reaction was carried out with 1 µmol of pincer Pd(II) complex **3a** and 5000 equiv. of MAO in 1,2-dichlorobenzene at 40 °C for 30 min, with a 98.7% monomer conversion rate and catalytic activities of 2.03 × 10^6^ g of PNB (mol of Pd)^−1^h^−1^ (Table 4, entry 1). Initially, we reduced the Al/Pd molar ratio to 3000; the monomer conversion rate had an obvious reduction (Table 4, entries 2–3). Subsequently, we screened the polymerization temperature (Table 4, entries 4–6). To our delight, the monomer conversion rate increased to 97.9% when the polymerization temperature was at 80 °C, while the color of the NB polymer was slightly dark (Table 4, entry 6). Therefore, we chose 40 °C as the optimal reaction temperature. Next, by shortening the reaction time to 6 min, the monomer conversion rate obviously reduced (57.2%, Table 4, entries 7–8). Reducing the amount of pre-catalyst to 0.8 μmol caused the monomer conversion rate and activity to decrease significantly (Table 4, entry 9). Therefore, Table 4, entry 1 was confirmed to be the optimal conditions. Under these conditions, we continued to explore the catalytic performance of the other PCN pincer Pd(II) complexes (Table 4, entries 10–16). Significantly, complex **3c** exhibited a superior monomer conversion rate (98.8%, Table 4, entry 11), whereas complexes **3d** and **4b** produced only trace amounts of polymer (Table 4, entries 12 and 15). Our investigation revealed that MAO, as a cocatalyst for norbornene polymerization, demonstrated superior catalytic activity and monomer conversion rates compared to EtAlCl_2_. However, the amount of MAO required was significantly higher than that of EtAlCl_2_.

### 2.3. Polymer Characterization

**Figure 2:**
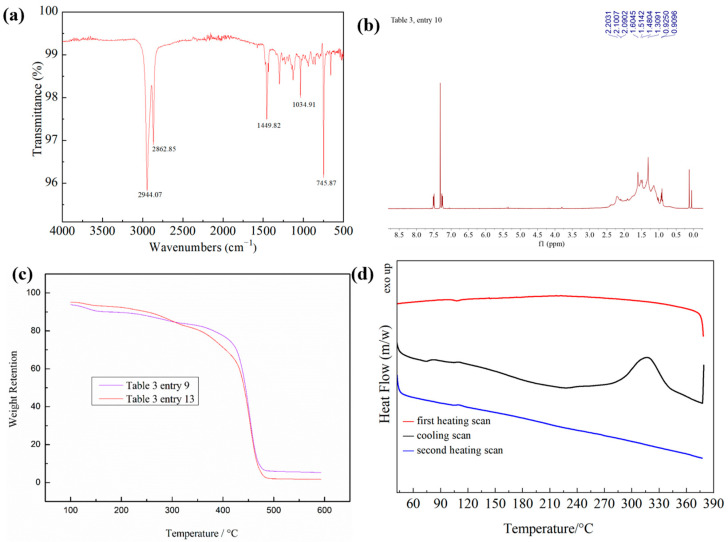
(**a**) FT-IR spectrum, (**b**) ^1^H NMR spectrum, and (**d**) DSC diagram of PNB obtained by **3a**/EtAlCl_2_ from Table 3, entry 10. (**c**) TGA thermograms of PNB obtained by **3a**–**b**/EtAlCl_2_ from Table 3, entries 9 and 13.

The synthesized polymers were characterized to determine their microstructure. First, the FT-IR spectrum of PNB revealed that there are no traces of (-C=C-) double bonds at 1600–1700 cm^−1^ (Figure 2a), and their ^1^H NMR spectra showed no peak around 5.0–6.0 ppm (Figure 2b), suggesting the presence of vinyl-type polynorbornene [19,48]. According to the thermogravimetric analysis (TGA) thermogram, the thermal stability of the polymer was as high as 400 °C (Figure 2c), and the polymer did not undergo a glass transition at 380 °C according to the analysis of the DSC diagram (Figure 2d). These results showed that the polymer had excellent thermal stability.

## 3. Materials and Methods

General Information: CNN pincer palladium(II) complexes **1** [36] and **2** [37], as well as PCN pincer palladium(II) complex **3** [38], were prepared according to the literature methods. All other chemicals were used as purchased. ^1^H NMR, ^13^C{^1^H} NMR, and ^31^P{^1^H} NMR spectra were recorded on a Bruker DPX-400 spectrometer with CDCl_3_ as the solvent and TMS as an internal standard (Bruker Corporation, Switzerland, Germany). TGA and DSC data were obtained via TGA-55 and DSC-Q2000 thermal analyzers, respectively (waters, Milford, MA USA). IR spectra were recorded using the Spectrum Two FT-IR Spectrometer (PerkinElmer, Waltham, MA, USA). Elemental analysis of catalysts was performed using Vario EL-III elemental analyzer (Elementar Company, Langenselbold, Germany).

### 3.1. Synthesis

#### 3.1.1. Synthesis of *m*-Hydroxybenzaldimines **3a′**–**e′**

Compounds **3a′**–**e′** were synthesized according to the procedure that we previously reported [38]. A mixture of *m*-hydroxybenzaldehyde (610.6 mg, 5.0 mmol) and primary amine (5.5 mmol) in toluene (20 mL) was refluxed for 10 h in the presence of activated Al_2_O_3_ (6.0 mmol) under an Ar atmosphere. After cooling, filtration, and evaporation, the residue was purified through flash column chromatography on a silica gel to afford the corresponding PCN pincer complexes **3a′**–**e′**. The new compounds, **3a′** and **3e′,** were characterized as follows.

*3-(((2-(tert-butyl)phenyl)imino)methyl)phenol* (**3a′**): Brown liquid (2.3 g, 10 mmol, and a 91% yield based on 2-(*tert*-butyl)aniline). ^1^H NMR (400 MHz, CDCl_3_): *δ* 8.18 (s, 1H, CH=N); 7.40–7.36 (m, 3H, and Ar-H); 7.26 (t, *J* = 7.8 Hz, 1H, and Ar-H); 7.22–7.13 (m, 2H, and Ar-H); 6.92–6.89 (m, 1H, and Ar-H); 6.80 (dd, *J* = 1.7, 7.4 Hz, 1H, and Ar-H); and 1.42 (s, 9H, and C(CH_3_)_3_) ppm. ^13^C{^1^H} NMR (100 MHz, CDCl_3_): *δ* 158.9, 156.2, 151.5, 143.0, 138.1, 130.3, 127.3, 126.3, 125.9, 122.2, 119.8, 118.9, 114.8, 35.8, and 30.7 ppm. HRMS (positive ESI): [M + H]^+^ calculated for C_17_H_20_NO^+^: 254.1539 was found to be 254.1543.
*3-(((3,5-di-tert-butylphenyl)imino)methyl)phenol* (**3e′**): Pale-brown solid (687.0 mg, 2.5 mmol, and a 89% yield based on 3,5-di-*tert*-butylaniline). mp 144–145 °C. ^1^H NMR (400 MHz, CDCl_3_): *δ* 8.42 (s, 1H, and CH=N); 7.46–7.45 (m, 1H, and Ar-H); 7.43–7.31 (m, 3H, and Ar-H); 7.05 (d, 2H, *J* = 1.7 Hz, and Ar-H); 6.99–6.96 (m, 1H, and Ar-H); and 1.35 (s, 18H, and C(CH_3_)_3_) ppm. ^13^C{^1^H} NMR (100 MHz, CDCl_3_): *δ* 160.0, 156.2, 151.9, 137.7, 130.1, 122.2, 120.4, 118.8, 115.2, 114.1, 110.1, 35.0, and 31.5 ppm.

#### 3.1.2. Synthesis of PCN Pincer Palladium(II) Complexes **3a**–**e**

Complexes **3a**–**e** were synthesized according to the procedure that we previously reported [38]. To a stirred solution of **3a′**–**e′** (0.5 mmol) and triethylamine (85 μL, 0.55 mmol) in toluene (20 mL), diphenylchlorophosphine (121.3 mg, 0.55 mmol) under an Ar atmosphere was added. The mixture was refluxed for 3 h. PdCl_2_ (89 mg, 0.5 mmol) was then added, and the reaction mixture was refluxed for another 6 h. After cooling, filtration, and evaporation, the residue was purified via column chromatography on a silica gel with CH_2_Cl_2_/petroleum ether (10/1) to afford the corresponding PCN pincer complexes **3a**–**e**. Complexes **3a**–**e** were characterized as follows.

*2-{N-(2-tert-butylphenyl)imino}-6-(diphenylphosphinoxy)phenylchloropalladium(II)* (**3a**): Yellow solid (66.9 mg, 58% yield). mp 248–249 °C. ^1^H NMR (400 MHz, CDCl_3_): *δ* 8.16 (d, *J* = 4.9 Hz, 1H, and CH=N); 8.05–8.00 (m, 4H, and Ph-H); 7.50–7.47 (m, 7H, Ph-H, and Ar-H); 7.24–7.13 (m, 4H, and Ar-H); 7.04–6.97 (m, 2H, and Ar-H); 1.51 (s, 9H, and C(CH_3_)_3_) ppm. ^13^C{^1^H} NMR (100 MHz, CDCl_3_): *δ* 175.2 (d, *J* = 4.4 Hz); 162.2 (d, *J* = 9.4 Hz); 155.5, 148.4, 145.6, 141.3, and 133.0 (d, *J* = 55.2 Hz); 132.4, 132.2, 132.1, and 131.6 (d, *J* = 14.5 Hz); 128.9 (d, *J* = 11.8 Hz); 127.6, 126.9, 126.8, 126.5, 124.2, 123.2, and 115.5 (d, *J* = 17.6 Hz); 36.1; and 32.6 ppm. ^31^P{^1^H} NMR (162 MHz, CDCl_3_): *δ* 155.4 ppm. Anal. Calcd for C_29_H_27_ClNOPPd**^.^**0.5H_2_O: C, 59.30; H, 4.80; N, 2.38; Found: C, 59.05; H, 4.88; N, 2.15.
*2-{N-(2,6-diisopropylphenyl)imino}-6-(diphenylphosphinoxy)phenylchloropalladium(II)* (**3b**): Yellow solid (36.4 mg, 30% yield). mp 319–320 °C. ^1^H NMR (400 MHz, CDCl_3_): *δ* 8.09 (d, *J* = 5.4 Hz, 1H, and CH=N); 8.07–8.02 (m, 4H, and Ph-H); 7.50–7.44 (m, 6H, and Ph-H); 7.21–7.14 (m, 5H, and Ar-H); 7.04 (d, *J* = 7.6 Hz, 1H, and Ar-H); 3.30–3.23 (m, 2H, and CH(CH_3_)_2_); 1.36 (d, *J* = 6.8 Hz, 6H, and CH(CH_3_)_2_); and 1.17 (d, *J* = 6.9 Hz, 6H, and CH(CH_3_)_2_) ppm. ^13^C{^1^H} NMR (100 MHz, CDCl_3_): *δ* 176.1 (d, *J* = 4.4 Hz); 162.1 (d, *J* = 9.5 Hz); 155.6 (d, *J* = 2.3 Hz); 145.4, 144.3, 140.5, and 133.1 (d, *J* = 54.7 Hz); 132.2 (d, *J* = 2.3 Hz); 131.9 (d, *J* = 14.6 Hz); 128.9 (d, *J* = 12.4 Hz); 127.2, 127.0, 123.2, 123.1, and 115.6 (d, *J* = 17.5 Hz); 28.6; 24.2; and 23.1 ppm. ^31^P{^1^H} NMR (162 MHz, CDCl_3_): *δ* 154.8 ppm. Anal. Calcd for C_31_H_31_ClNOPPd**^.^**0.5H_2_O: C, 60.50; H, 5.24; N, 2.28. Found: C, 60.73; H, 5.23; N, 2.06.
*2-{N-(2,6-dimethylphenyl)imino}-6-(diphenylphosphinoxy)phenylchloropalladium(II)* (**3c**): Yellow solid (76.9 mg, 70% yield). mp 245–246 °C. ^1^H NMR (400 MHz, CDCl_3_): *δ* 8.09 (d, *J* = 5.4 Hz, 1H, and CH=N); 8.06–8.01 (m, 4H, and Ph-H); 7.49–7.44 (m, 6H, and Ph-H); 7.18–7.13 (m, 2H, and Ar-H); 7.09–7.03 (m, 4H, and Ar-H); and 2.33 (s, 6H, and CH_3_) ppm. ^13^C{^1^H} NMR (100 MHz, CDCl_3_): *δ* 176.8 (d, *J* = 4.6 Hz); 162.2 (d, *J* = 9.8 Hz); 155.3 (d, *J* = 2.2 Hz); 145.6, 144.5, 135.9, and 133.0 (d, *J* = 54.2 Hz); 132.2 (d, *J* = 2.5 Hz); 131.8 (d, *J* = 15.1 Hz); 129.6 and 128.9 (d, *J* = 11.8 Hz); 128.8, 127.0, 123.0, and 115.5 (d, *J* = 16.8 Hz); 21.0; and 19.0 ppm. ^31^P{^1^H} NMR (162 MHz, CDCl_3_): *δ* 154.5 ppm. Anal. Calcd for C_27_H_23_ClNOPPd: C, 58.93; H, 4.21; N, 2.55. Found: C, 58.66; H, 4.27; N, 2.33.
*2-{N-(2,4,6-trimethylphenyl)imino}-6-(diphenylphosphinoxy)phenylchloropalladium(II)* (**3d**): Yellow solid (69.8 mg, 62% yield). mp 319–320 °C. ^1^H NMR (400 MHz, CDCl_3_): *δ* 8.07 (d, *J* = 5.5 Hz, 1H, and CH=N); 8.06–8.01 (m, 4H, and Ph-H); 7.52–7.44 (m, 6H, and Ph-H); 7.17–7.12 (m, 2H, and Ar-H); 7.04–7.02 (m, 1H, and Ar-H); 6.91 (s, 2H, and Ar-H); and 2.30–2.29 (m, 9H, and CH_3_) ppm. ^13^C{^1^H} NMR (100 MHz, CDCl_3_): *δ* 176.9 (d, *J* = 4.4 Hz); 162.2 (d, *J* = 9.6 Hz); 155.3 (d, *J* = 1.7 Hz); 145.6, 144.5, 136.0, and 133.0 (d, *J* = 54.5 Hz); 132.2 (d, *J* = 2.4 Hz); 131.8 (d, *J* = 14.8 Hz); 129.7 and 128.9 (d, *J* = 11.9 Hz); 128.8, 127.0, 123.1, and 115.5 (d, *J* = 17.2 Hz); 21.0; and 19.0 ppm. ^31^P{^1^H} NMR (162 MHz, CDCl_3_): *δ* 154.4 ppm. Anal. Calcd for C_28_H_25_ClNOPPd**^.^**0.5H_2_O: C, 58.65; H, 4.57; N, 2.44. Found: C, 58.91; H, 4.52; N, 2.20.
*2-{N-(3,5-di-tert-butylphenyl)imino}-6-(diphenylphosphinoxy)phenylchloropalladium(II)* (**3e**): Yellow solid (57.0 mg, 45% yield). mp 247–248 °C. ^1^H NMR (400 MHz, CDCl_3_): *δ* 8.36 (d, *J* = 4.6 Hz, 1H, and CH=N); 8.08–8.03 (m, 4H, and Ph-H); 7.50–7.44 (m, 6H, and Ph-H); 7.40–7.39 (m, 3H, and Ar-H); 7.22 (d, *J* = 7.4 Hz, 1H, and Ar-H); 7.16–7.12 (m, 1H, and Ar-H); 7.00 (d, *J* = 8.0 Hz, 1H, and Ar-H); and 1.37 (s, 18H, and C(CH_3_)_3_) ppm. ^13^C{^1^H} NMR (100 MHz, CDCl_3_): *δ* 172.1 (d, *J* = 4.0 Hz); 162.2 (d, *J* = 9.4 Hz); 154.4, 151.5, 147.5, 146.2, and 132.8 (d, *J* = 55.6 Hz); 132.2 (d, *J* = 2.5 Hz); 131.8 (d, *J* = 14.4 Hz); 128.9 (d, *J* = 11.9 Hz); 127.0, 123.2, 122.1, 118.0, and 115.2 (d, *J* = 17.7 Hz); 35.2; and 31.5 ppm. ^31^P{^1^H} NMR (162 MHz, CDCl_3_): *δ* 153.7 ppm. Anal. Calcd for C_33_H_35_ClNOPPd**^.^**0.5H_2_O: C, 61.59; H, 5.64; N, 2.18. Found: C, 62.05; H, 5.65; N, 1.97.

### 3.2. General Procedure for Norbornene Polymerization

Under an argon atmosphere, norbornene (1.03 g) was dissolved in 5 mL of 1,2-dichlorobenzene; then, the exact amount of the EtAlCl_2_, Et_2_AlCl, or MAO solution was added. Next, to keep the mixture at the desired temperature for 3 min, the appropriate amount of the pincer Pd(II) complex dissolved in 1,2-dichlorobenzene was injected into the flask. The reaction mixture was stirred for 30 min, and 10% acidic ethanol was added to terminate the reaction. The PNB was washed with ethanol and dried at 80 °C in vacuo to a constant weight. In all the polymerization processes, the total reaction volume was 10 mL, which can be achieved by changing the amount of 1,2-dichlorobenzene added as necessary.

## 4. Conclusions

In summary, we synthesized a series of CNN and PCN pincer Pd(II) complexes, and these complexes were applied to the polymerization of norbornene. With the cocatalyst Et_2_AlCl, the CNN pincer Pd(II) complexes exhibited higher catalytic activities (9.6 × 10^6^ g of PNB (mol of Pd)^−1^h^−1^) and a higher monomer conversion rate (99.5%) compared to EtAlCl_2_. In the presence of the cocatalyst MAO, the PCN pincer Pd(II) complexes exhibited higher catalytic activities (5.9 × 10^6^ g of PNB (mol of Pd)^−1^h^−1^) and monomer conversion rates (98.8%) compared to the cocatalyst EtAlCl_2_. The monomer conversion rates (65.7–79.2%) and catalytic activities (4.06–4.89 × 10^6^ g of PNB (mol of Pd)^−1^h^−1^) of the polymer given by the CNN pincer Pd(II) complexes were greater than those (monomer conversion rates: 25.9–85.3%; catalytic activities: 0.53–1.76 × 10^6^ g of PNB (mol of Pd)^−1^h^−1^) given by the PCN pincer Pd(II) complexes.

## Figures and Tables

**Table 1:** Vinyl polymerization of norbornene with CNN pincer Pd(II) complexes activated by EtAlCl_2_ *^a^*.

Entry	Cat.	Temp. (°C)	*t* (min)	[Cat.] (μmol)	Al/Pd	PNB (g)	Conv. (%)	Activity *^b^*
1 *^c^*	―	40	30	―	―	0	0	0
2	**1a**	40	30	1.0	2000	0.7513	72.94	1.5026
3	**1a**	40	30	1.0	2500	0.6842	66.43	1.3684
4	**1a**	40	30	1.0	1500	0.7328	71.15	1.4656
5	**1a**	0	30	1.0	2000	0.2606	25.30	0.5212
6	**1a**	20	30	1.0	2000	0.8273	80.32	1.6546
7	**1a**	60	30	1.0	2000	0.7436	72.19	1.4872
8	**1a**	80	30	1.0	2000	0.7558	73.38	1.5116
9	**1a**	20	20	1.0	2000	0.8197	79.58	2.4591
10	**1a**	20	10	1.0	2000	0.8153	79.16	4.8918
11	**1a**	20	5	1.0	2000	0.7683	74.59	9.2196
12	**1a**	20	10	1.2	2000	0.8072	78.37	4.0360
13	**1a**	20	10	0.8	2000	0.7264	70.52	5.4480
14	**1b**	20	10	1.0	2000	0.7032	68.27	4.2192
15	**1c**	20	10	1.0	2000	0.6767	65.70	4.0602
16	**1d**	20	10	1.0	2000	0.6907	67.06	4.1442
17	**1e**	20	10	1.0	2000	0.7018	68.14	4.2108
18	**2a**	20	10	1.0	2000	0.7172	69.63	4.3032
19	**2b**	20	10	1.0	2000	0.7558	73.38	4.5348
20	**2c**	20	10	1.0	2000	0.7973	77.41	4.7838

*^a^* Polymerization conditions: solvent, 1,2-dichlorobenzene; *V*_total_, 10 mL; norbornene, 1.03 g; EtAlCl_2_, 1.1 M in hexane. *^b^* In units of 10^6^ g of PNB (mol of Pd)^−1^ h^−1^. *^c^* In units of 2000 μmol of EtAlCl_2_.

**Table 2:** Vinyl polymerization of norbornene with CNN pincer Pd(II) complexes activated by Et_2_AlCl *^a^*.

Entry	Cat.	Temp. (°C)	*t* (min)	[Cat.] (μmol)	Al/Pd	PNB (g)	Conv. (%)	Activity *^b^*
1	**1a**	40	8	1.0	2000	0.9654	93.73	7.2405
2	**1a**	40	8	1.0	1500	0.9876	95.88	7.4070
3	**1a**	40	8	1.0	1000	0.5732	55.65	4.2990
4	**1a**	40	8	1.0	500	0.4230	41.07	3.1725
5	**1a**	0	8	1.0	1500	0.9281	90.11	6.9608
6	**1a**	20	8	1.0	1500	1.0253	99.54	7.6898
7	**1a**	60	8	1.0	1500	0.1279	12.42	0.9593
8	**1a**	80	8	1.0	1500	0.0539	5.23	0.4043
9	**1a**	20	8	0.8	1500	0.8976	87.15	8.4150
10	**1a**	20	6	1.0	1500	0.9597	93.17	9.5970
11	**1a**	20	10	1.0	1500	0.9362	90.89	5.6172
12	**1b**	20	8	1.0	1500	1.0233	99.35	7.6748
13	**1c**	20	8	1.0	1500	1.0025	97.33	7.6688
14	**1d**	20	8	1.0	1500	0.9663	93.82	7.2473
15	**1e**	20	8	1.0	1500	0.9935	96.46	7.4513
16	**2a**	20	8	1.0	1500	0.9001	87.39	6.7508
17	**2b**	20	8	1.0	1500	0.9268	89.98	6.9510
18	**2c**	20	8	1.0	1500	1.0051	97.58	7.5383

*^a^* Polymerization conditions: solvent, 1,2-dichlorobenzene; *V*_total_, 10 mL; norbornene, 1.03 g; Et_2_AlCl, 1.0 M in hexane. *^b^* In units of 10^6^ g of PNB (mol of Pd)^−1^ h^−1^.

**Table 3:** Vinyl polymerization of norbornene with PCN pincer Pd(II) complexes activated by EtAlCl_2_ *^a^*.

Entry	Cat.	Temp. (°C)	*t* (min)	[Cat.] (μmol)	Al/Pd	PNB (g)	Conv. (%)	Activity *^b^*
1	**3a**	40	10	1.0	1000	0.2642	25.65	1.5852
2	**3a**	40	10	1.0	1500	0.2065	20.05	1.2390
3	**3a**	40	10	1.0	2000	0.7095	68.89	4.2570
4	**3a**	40	10	1.0	2500	0.5676	55.11	3.4056
5	**3a**	0	10	1.0	2000	0.6235	60.53	1.2470
6	**3a**	20	10	1.0	2000	0.6587	63.95	1.3174
7	**3a**	60	10	1.0	2000	0.4025	39.08	2.4150
8	**3a**	80	10	1.0	2000	0.5705	55.39	3.4230
9	**3a**	40	30	1.0	2000	0.8788	85.32	1.7576
10	**3a**	40	60	1.0	2000	0.8827	85.70	0.8827
11	**3a**	40	30	1.2	2000	0.8735	84.81	1.4558
12	**3a**	40	30	0.8	2000	0.7243	70.32	1.4486
13	**3b**	40	30	1.0	2000	0.6421	62.34	1.2842
14	**3c**	40	30	1.0	2000	0.5068	49.20	1.0136
15	**3d**	40	30	1.0	2000	0.3625	35.19	0.7250
16	**3e**	40	30	1.0	2000	0.2663	25.85	0.5326
17	**4a**	40	30	1.0	2000	0.6097	59.19	1.2194
18	**4b**	40	30	1.0	2000	0.6259	66.47	1.2518
19	**4c**	40	30	1.0	2000	0.4715	45.78	0.9430

*^a^* Polymerization conditions: solvent, 1,2-dichlorobenzene; *V*_total_, 10 mL; norbornene, 1.03 g; EtAlCl_2_, 1.1 M in hexane. *^b^* In units of 10^6^ g of PNB (mol of Pd)^−1^ h^−1^.

**Table 4:** Vinyl polymerization of norbornene with PCN pincer Pd(II) complexes activated by MAO *^a^.*

Entry	Cat.	Temp. (°C)	*t* (min)	[Cat.] (μmol)	Al/Pd	PNB (g)	Conv. (%)	Activity *^b^*
1	**3a**	40	30	1.0	5000	1.0163	98.67	2.0326
2	**3a**	40	30	1.0	4000	0.6068	58.91	1.2136
3	**3a**	40	30	1.0	3000	0.4348	42.21	0.8696
4	**3a**	20	30	1.0	5000	0.8762	85.07	1.7524
5	**3a**	60	30	1.0	5000	0.7411	71.95	1.4822
6	**3a**	80	30	1.0	5000	1.0083	97.89	2.016
7	**3a**	40	15	1.0	5000	0.7091	68.84	2.8364
8	**3a**	40	6	1.0	5000	0.5889	57.17	5.8890
9	**3a**	40	30	0.8	5000	0.6063	58.86	1.5158
10	**3b**	40	30	1.0	5000	0.2369	23.00	0.4738
11	**3c**	40	30	1.0	5000	1.0172	98.76	2.0344
12	**3d**	40	30	1.0	5000	trace	/	/
13	**3e**	40	30	1.0	5000	0.5699	55.33	1.1398
14	**4a**	40	30	1.0	5000	0.1045	10.15	0.2090
15	**4b**	40	30	1.0	5000	trace	/	/
16	**4c**	40	30	1.0	5000	0.2375	23.06	0.4750

*^a^* Polymerization conditions: solvent, 1,2-dichlorobenzene; *V*_total_, 10 mL; norbornene, 1.03 g; MAO, 10 wt.% solution in toluene. *^b^* In units of 10^6^ g of PNB (mol of Pd)^−1^ h^−1^.

## Data Availability

The data presented in this study are available upon request from the corresponding author.

## Supplementary Materials

The following supporting information can be downloaded at https://www.mdpi.com/article/10.3390/molecules30071530/s1, Table S1. Crystal Structure Determination for **3a**. Scheme S1. Possible mechanism for EtAlCl_2_-activated norbornene polymerization. Excess EtAlCl_2_ first alkylates the **S1a** and then abstracts the ethyl group form cationic species. Due to the instability of cationic species, an asymmetric binuclear complex **S1b** with a neutral molecule was formed through the bridge forms. A norbornene molecule firstly coordinate to the Pd^+^ in **S1c**, and then the ethyl group attacks and inserts into the double bond of norbornene to generate **S1d**. After the reaction, the cation center transfers to the another Pd atom in **S1d**. A new norbornene molecule coordinate to the Pd^+^ in **S1e**, and then the norbornenyl group attacks and inserts into the double bond of norbornene to generate **S1f**. Continuous coordination and insertion of norbornene alternatively on the two Pd centers of binuclear complexes produce the resultant polynorbornene. Table S2. Comparison of Catalytic properties of the CNN and PCN pincer Pd(II) complexes with Previously Reported Pd(II) Complexes for Norbornene Polymerization. Figure S1. ^1^H NMR (400 MHz, CDCl_3_) spectrum of **3a′**. Figure S2. ^13^C{^1^H}NMR (100 MHz, CDCl_3_) spectrum of **3a′**. Figure S3. ^1^H NMR (400 MHz, CDCl_3_) spectrum of **3e′**. Figure S4. ^13^C{^1^H}NMR (100 MHz, CDCl_3_) spectrum of **3e′**. Figure S5. ^1^H NMR (400 MHz, CDCl_3_) spectrum of **3a**. Figure S6. ^13^C{^1^H}NMR (100 MHz, CDCl_3_) spectrum of **3a**. Figure S7. ^31^P{^1^H}NMR (162 MHz, CDCl_3_) spectrum of **3a**. Figure S8. ^1^H NMR (400 MHz, CDCl_3_) spectrum of **3b**. Figure S9. ^13^C{^1^H}NMR (100 MHz, CDCl_3_) spectrum of **3b**. Figure S10. ^31^P{^1^H}NMR (162 MHz, CDCl_3_) spectrum of **3b**. Figure S11. ^1^H NMR (400 MHz, CDCl_3_) spectrum of **3c**. Figure S12. ^13^C{^1^H}NMR (100 MHz, CDCl_3_) spectrum of **3c**. Figure S13. ^31^P{^1^H}NMR (162 MHz, CDCl_3_) spectrum of **3c**. Figure S14. ^1^H NMR (400 MHz, CDCl_3_) spectrum of **3d**. Figure S15. ^13^C{^1^H}NMR (100 MHz, CDCl_3_) spectrum of **3d**. Figure S16. ^31^P{^1^H}NMR (162 MHz, CDCl_3_) spectrum of **3d**. Figure S17. ^1^H NMR (400 MHz, CDCl_3_) spectrum of **3e**. Figure S18. ^13^C{^1^H}NMR (100 MHz, CDCl_3_) spectrum of **3e**. Figure S19. ^31^P{^1^H}NMR (162 MHz, CDCl_3_) spectrum of **3e**. Figure S20. IR spectrum of PNB obtained by **1a**/EtAlCl_2_ (Table 1, entry 9). Figure S21. IR spectrum of PNB obtained by **2c**/EtAlCl_2_ (Table 1, entry 19). Figure S22. IR spectrum of PNB obtained by **1a**/Et_2_AlCl (Table 2, entry 6). Figure S23. IR spectrum of PNB obtained by **1c**/Et_2_AlCl (Table 2, entry 13). Figure S24. IR spectrum of PNB obtained by **4b**/EtAlCl_2_ (Table 3 entry 18). Figure S25. IR spectrum of PNB obtained by **3a**/MAO (Table 4, entry 1). Figure S26. IR spectrum of PNB obtained by **4c**/MAO (Table 4, entry 16). Figure S27. DSC thermograms of PNB obtained by **1a**/EtAlCl_2_ (Table 1, entry 9). Figure S28. DSC thermograms of PNB obtained by **2c**/EtAlCl2 (Table 1, entry 19). Figure S29. DSC thermograms of PNB obtained by **1a**/Et_2_AlCl (Table 2, entry 6). Figure S30. DSC thermograms of PNB obtained by **1c**/Et2AlCl (Table 2, entry 13). Figure S31. DSC thermograms of PNB obtained by **4c**/EtAlCl2 (Table 3, entry 19). Figure S32. DSC thermograms of PNB obtained by **3a**/MAO (Table 4, entry 1). Figure S33. DSC thermograms of PNB obtained by **4c**/MAO (Table 4, entry 16). Figure S34. TGA of PNB. Figure S35. TGA of PNB. Figure S36. TGA of PNB.
