# Supplementary material for: Synthesis, Characterization, and Comparative Study on Norbornene Polymerization of CNN and PCN Pincer Palladium Complexes
Source: Molecules. 2025 Mar 30;30(7):1530. doi: 10.3390/molecules30071530 (PMC11990364; doi:10.3390/molecules30071530)
Supplement: Supplementary file 1 [file molecules-30-01530-s001.zip › molecules-3547431-supplementary.pdf]

Supporting Information

Synthesis, Characterization, and Comparative Study on Norbornene Polymerization of CNN and PCN Pincer Palladium Complexes

Huizhu Wang †, Jin-Kui Liu †, Yi-Dong Wang, Xin-Qi Hao, Mao-Ping Song, Jun-Fang Gong * and Hui Jiang *

College of Chemistry, Pingyuan Laboratory, Zhengzhou University, Zhengzhou 450001, China.

E-mail: gongjf@zzu.edu.cn (J.-F. Gong) or jiangh@zzu.edu.cn (H. Jiang).

X-ray Diffraction Studies of Complex **3a**........S2

The possible norbornene polymerization mechanism........S3

The comparison with reported catalysts........S4

NMR Spectra........S5

IR Spectra of norbornene homopolymers........S14

DSC data of norbornene homopolymers........S18

TGA data of norbornene homopolymers........S21

X-ray Diffraction Studies of Complex 3a

Table S1. Crystal Structure Determination for **3a**

Empirical formula	$C_{29}H_{27}ClNOPPd$
Formula weight	578.33
Temperature/K	293(2)
Crystal system	monoclinic
Space group	C2/c
a/Å	31.7386(3)
b/Å	10.00330(6)
c/Å	33.1105(3)
α/ °	90
β/°	94.2381(8)
γ/°	90
Volume/Å^3	10483.55(14)
Z	16
ρ_{calc} g/cm^3	1.466
μ/mm^{-1}	7.390
F(000)	4704.0
Crystal size/mm^3	0.16 × 0.12 × 0.1
Radiation	CuKα (λ = 1.54184)
2θ range for data collection/ °	7.446 to 134.152
Index ranges	$-37 \leq h \leq 37$, $-11 \leq k \leq 9$, $-38 \leq l \leq 39$
Reflections collected	45533
Independent reflections	9333 [R_{int} = 0.0435, R_{sigma} = 0.0309]
Data/restraints/parameters	9333/114/585
Goodness-of-fit on F^2	1.022
Final R indexes [I>=2σ (I)]	R_1 = 0.0419, wR_2 = 0.1085
Final R indexes [all data]	R_1 = 0.0503, wR_2 = 0.1152
Largest diff. peak/hole / e Å^{-3}	0.99/-0.53
CCDC number	2205081

The possible norbornene polymerization mechanism

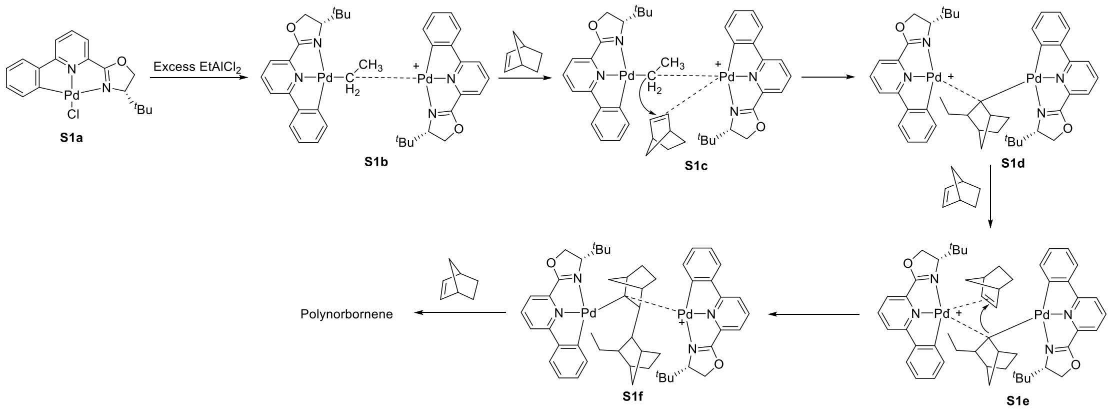

Scheme S1. Possible mechanism for $EtAlCl_2$-activated norbornene polymerization. Excess $EtAlCl_2$ first alkylates the **S1a** and then abstracts the ethyl group form cationic species. Due to the instability of cationic species, an asymmetric binuclear complex **S1b** with a neutral molecule was formed through the bridge forms. A norbornene molecule firstly coordinate to the Pd^+ in **S1c**, and then the ethyl group attacks and inserts into the double bond of norbornene to generate **S1d**. After the reaction, the cation center transfers to the another Pd atom in **S1d**. A new norbornene molecule coordinate to the Pd^+ in **S1e**, and then the norbornenyl group attacks and inserts into the double bond of norbornene to generate **S1f**. Continuous coordination and insertion of norbornene alternatively on the two Pd centers of binuclear complexes produce the resultant polynorbornene.

The comparison with reported catalysts

The Previously reported catalytic properties of palladium(II) catalysts for norbornene were summarized in Table S2. The comparative data demonstrate that our catalysts had similar or competitive activity. Among them, the catalytic activity of NCP pincer Pd(II) complex **A** reached 8.78 $\times 10^6$ g of PNB (mol of Pd)$^{-1}$h^{-1} under the activation of 8000 equiv. MAO, while the conversion rate of norbornene monomer was only 87% (Table S2, entry 1). Although PNN pincer Pd(II) complex **B** and NNN pincer Pd(II) complex **D** had higher catalytic activity and monomer conversion, they required the use of more expensive cocatalyst MAO or MMAO, which obviously increases the production cost of polynorbornene (Table S2, entries 2 and 4). In contrast, our CNN pincer Pd(II) complexes **1a** and **2c** only used a cheaper and smaller amount of cocatalyst Et_2AlCl, which can achieve almost quantitative conversion of norbornene, and its polymerization activity can reach 7.54 $\times$ 10^6 g of PNB (mol of Pd)$^{-1}$h^{-1} (Table S2, entries 6 and 7). These results showed the high efficiency and economy of CNN pincer Pd(II) complexes.

Table S2. Comparison of Catalytic properties of the CNN and PCN pincer Pd(II) complexes with Previously Reported Pd(II) Complexes for Norbornene Polymerization

Entry	Cat.	Cocat.	Al/Pd	Conv. (%)	Activity[a]	Reference
1	**A**	MAO	8000	87	8.78	[1]
2	**B**	MMAO	1000	99	59.00	[2]
3	**C**	$EtAlCl_2$	1000	24	14.00	[2,3]
4	**D**	MAO	8000	100	360.00	[4]
5	**E**	MAO	15000	76	11.50	[5]
6	**1a**	Et_2AlCl	1500	100	7.69	This work
7	**2c**	Et_2AlCl	1500	98	7.54	This work
8	**3a**	MAO	5000	99	2.03	This work
9	**4a**	$EtAlCl_2$	2000	59	1.22	This work

[a]In units of 10^6 g of PNB (mol of Pd)$^{-1}$ h^{-1}.

NMR Spectra

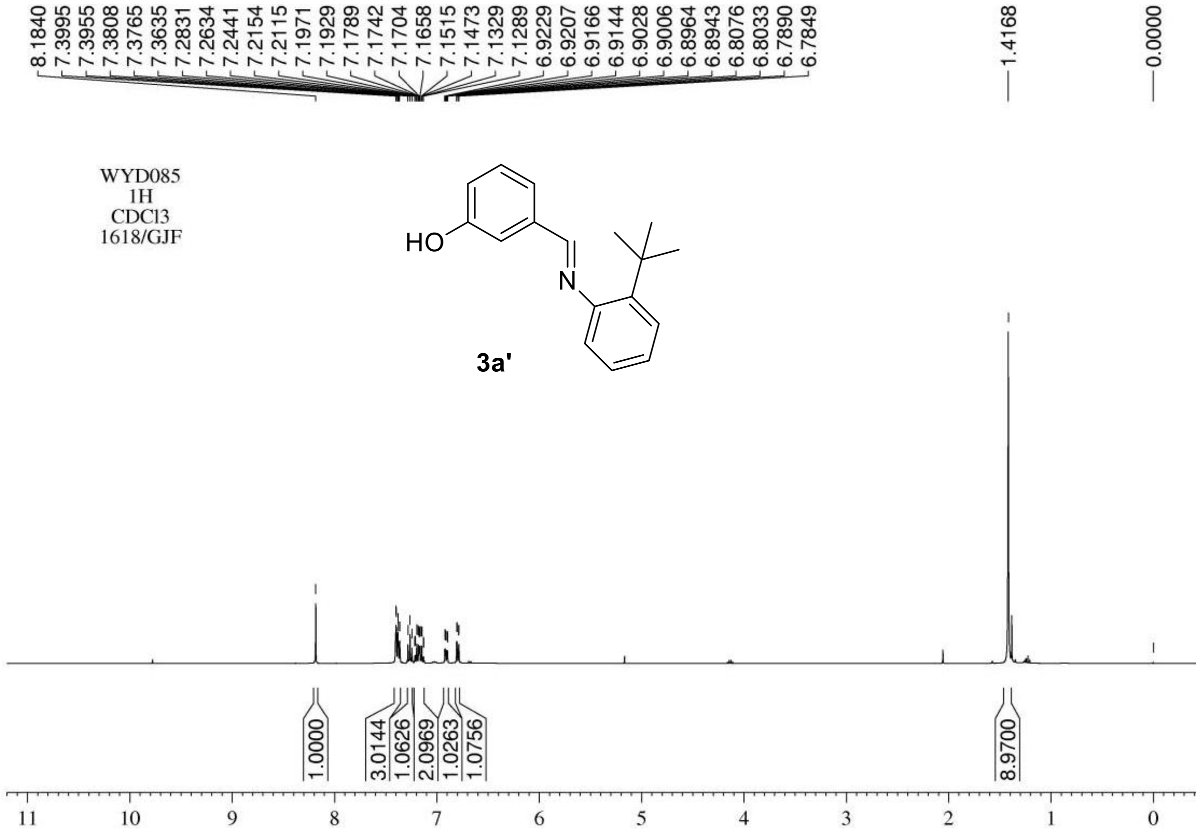

Figure S1. ^{1}H NMR (400 MHz, $CDCl_3$) spectrum of **3a'**

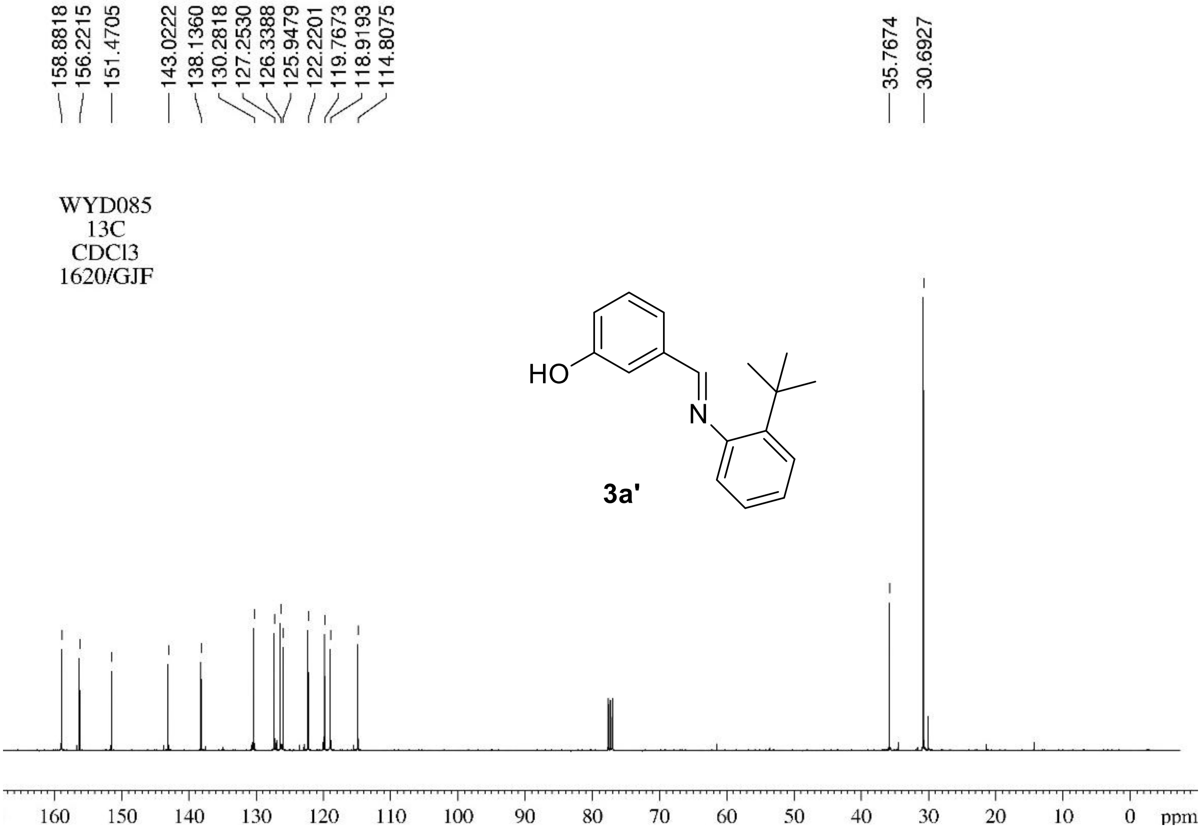

Figure S2. ^{13}C{^{1}H} NMR (100 MHz, $CDCl_3$) spectrum of **3a'**

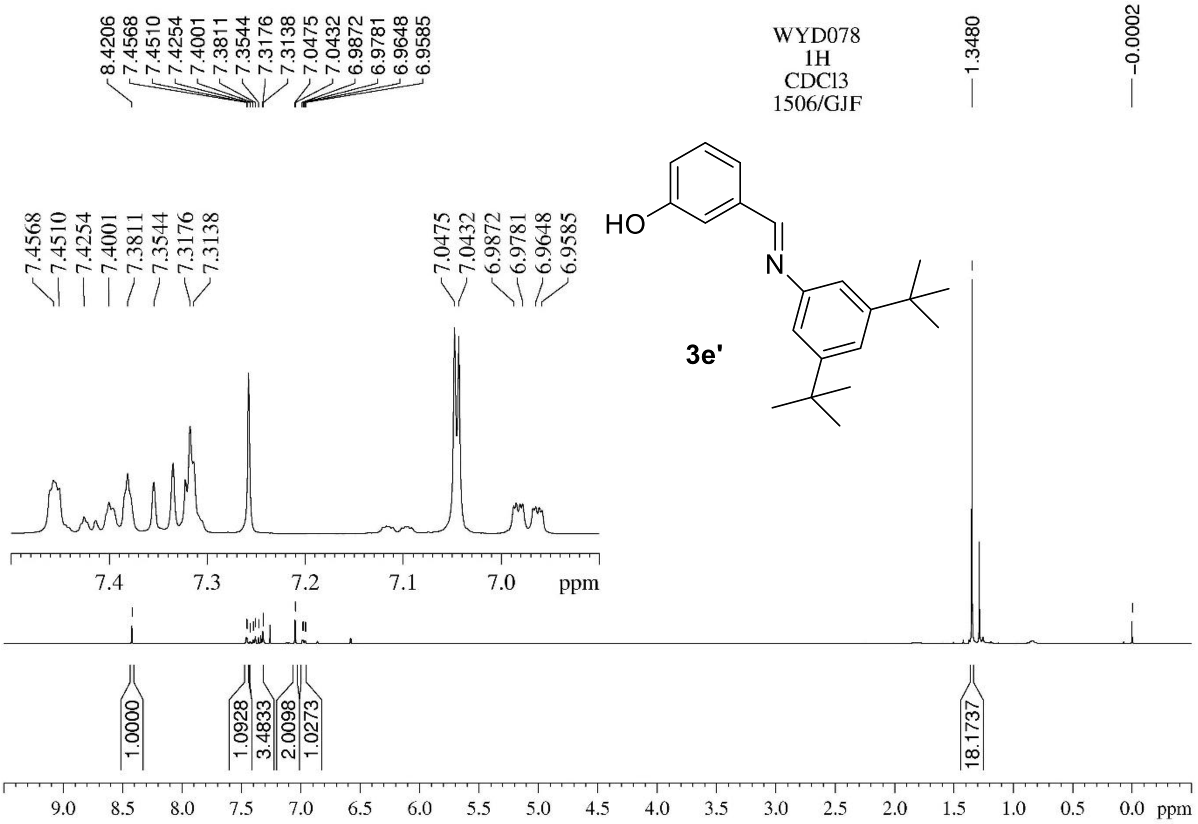

Figure S3. ^{1}H NMR (400 MHz, $CDCl_3$) spectrum of **3e'**

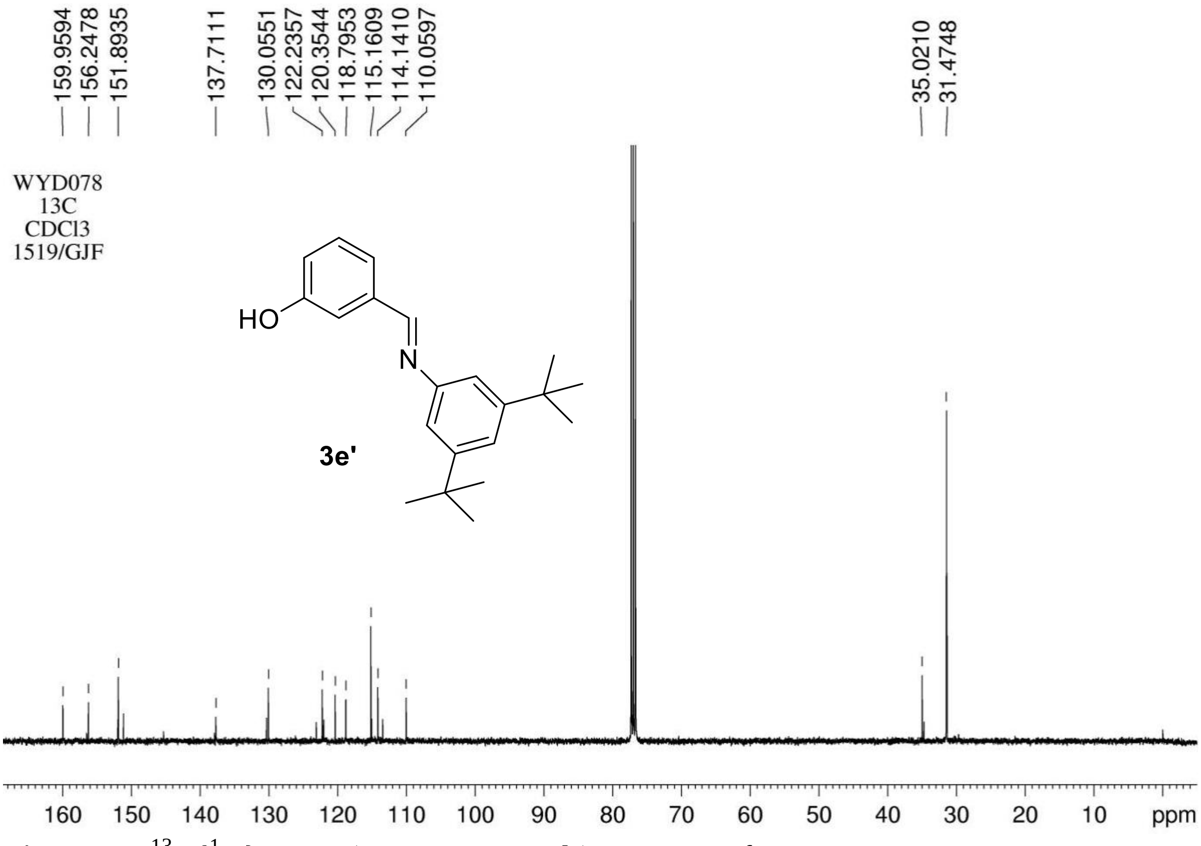

Figure S4. $^{13}C\{^{1}H\}$ NMR (100 MHz, $CDCl_3$) spectrum of **3e'**

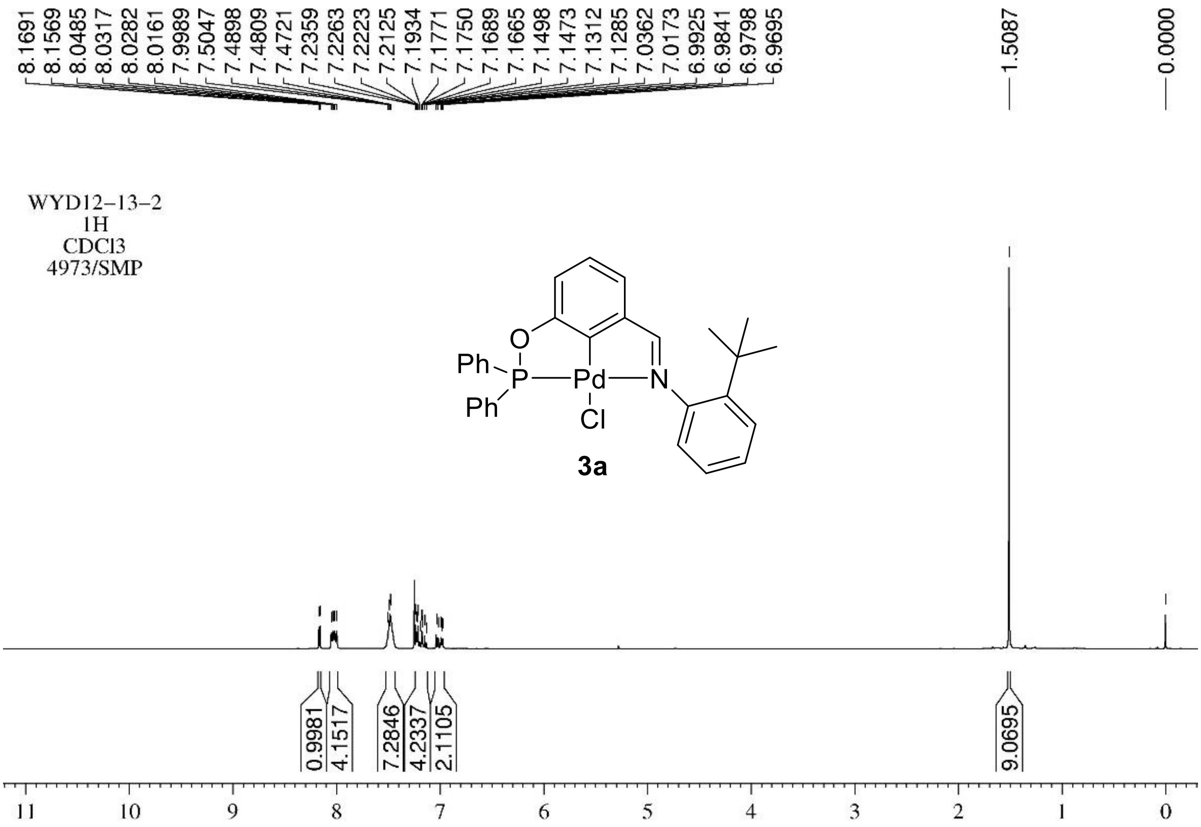

Figure S5. ^{1}H NMR (400 MHz, $CDCl_3$) spectrum of **3a**

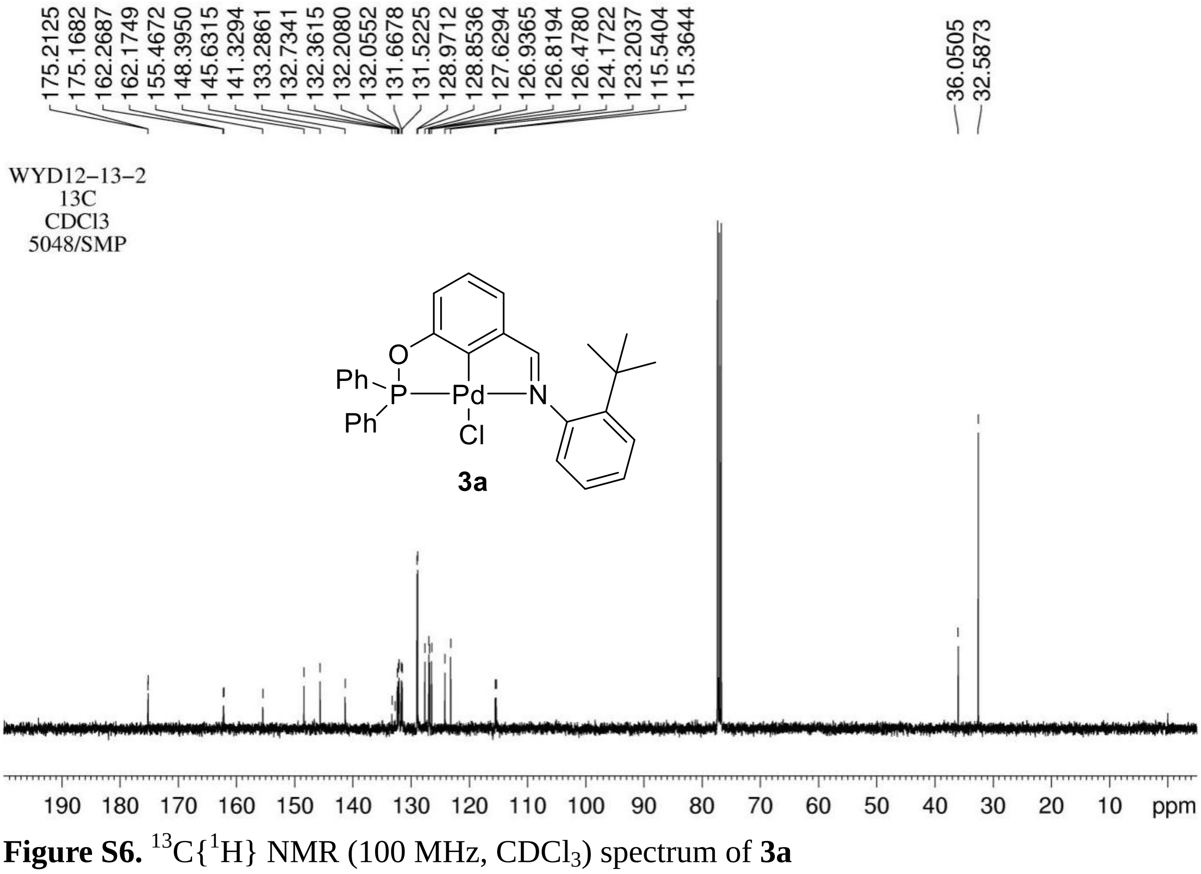

Figure S6. $^{13}C\{^{1}H\}$ NMR (100 MHz, $CDCl_3$) spectrum of **3a**

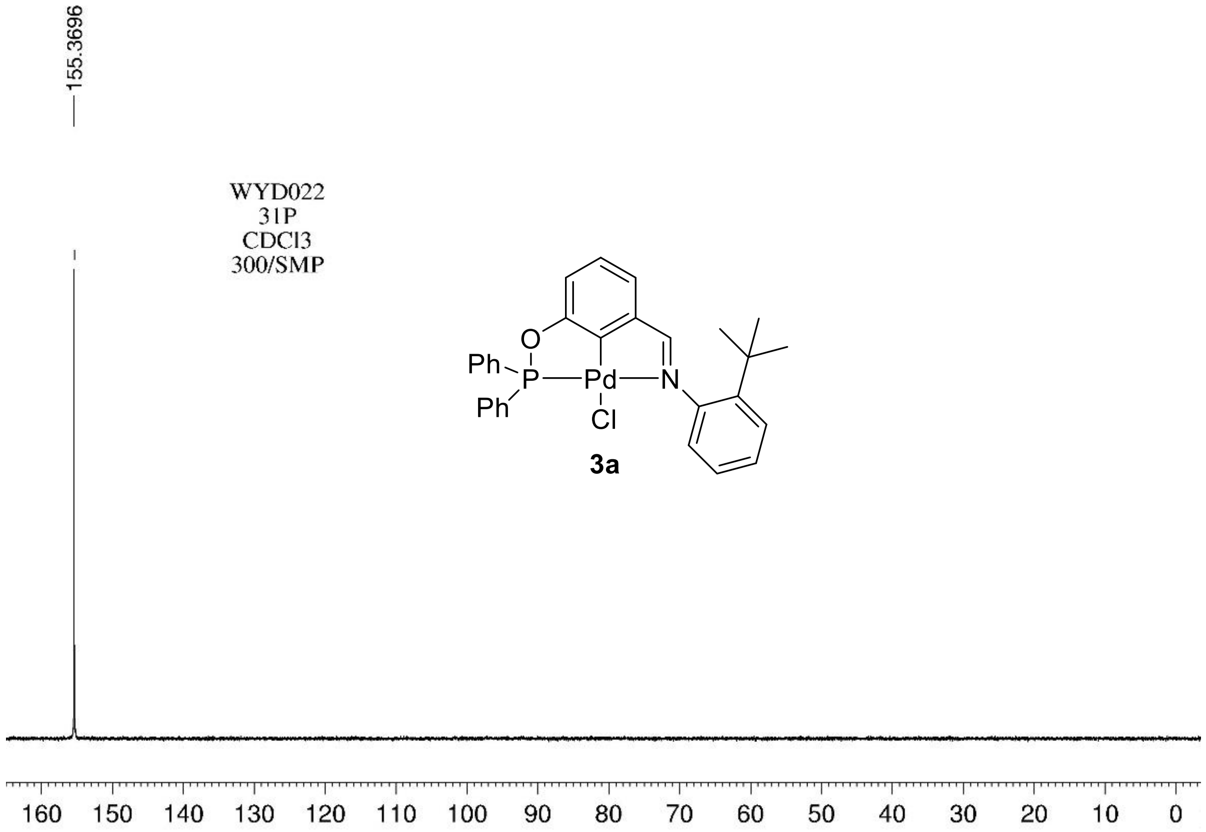

Figure S7. $^{31}P\{^{1}H\}$ NMR (162 MHz, $CDCl_3$) spectrum of **3a**

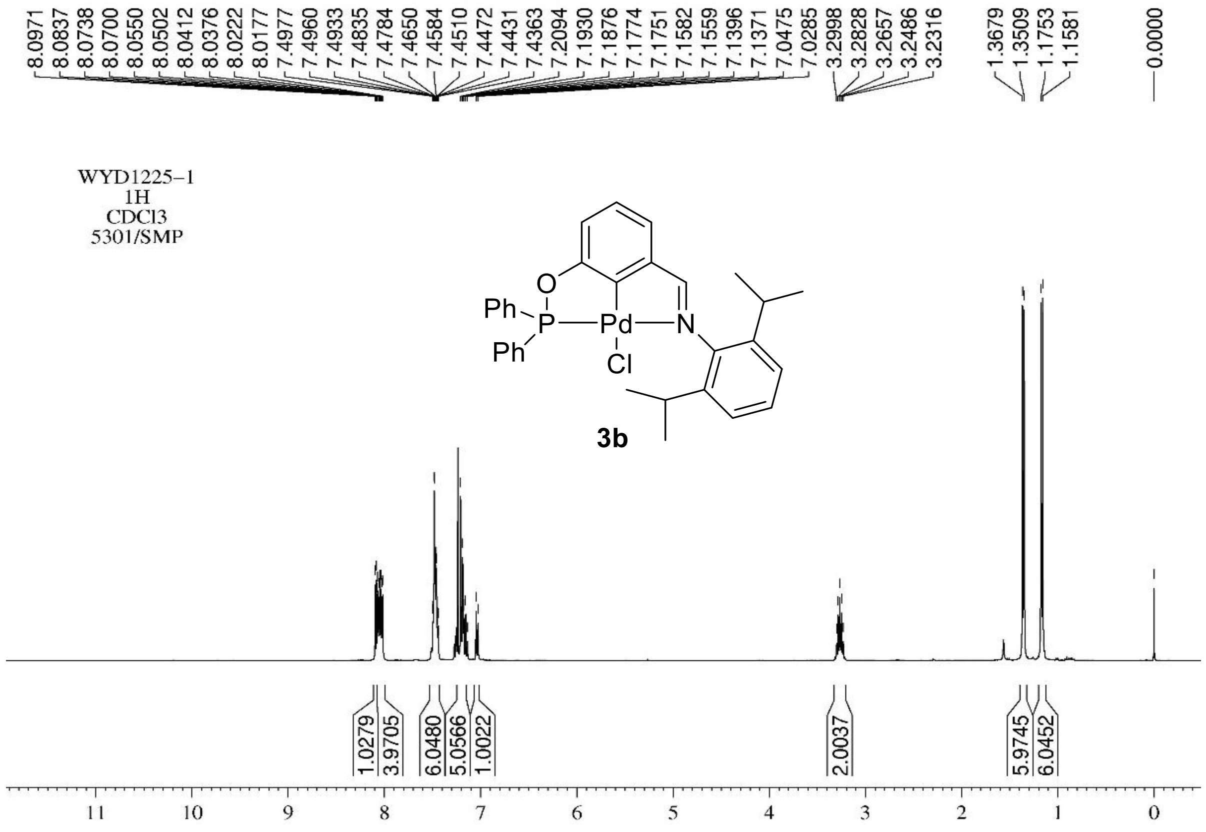

Figure S8. ^{1}H NMR (400 MHz, $CDCl_3$) spectrum of **3b**

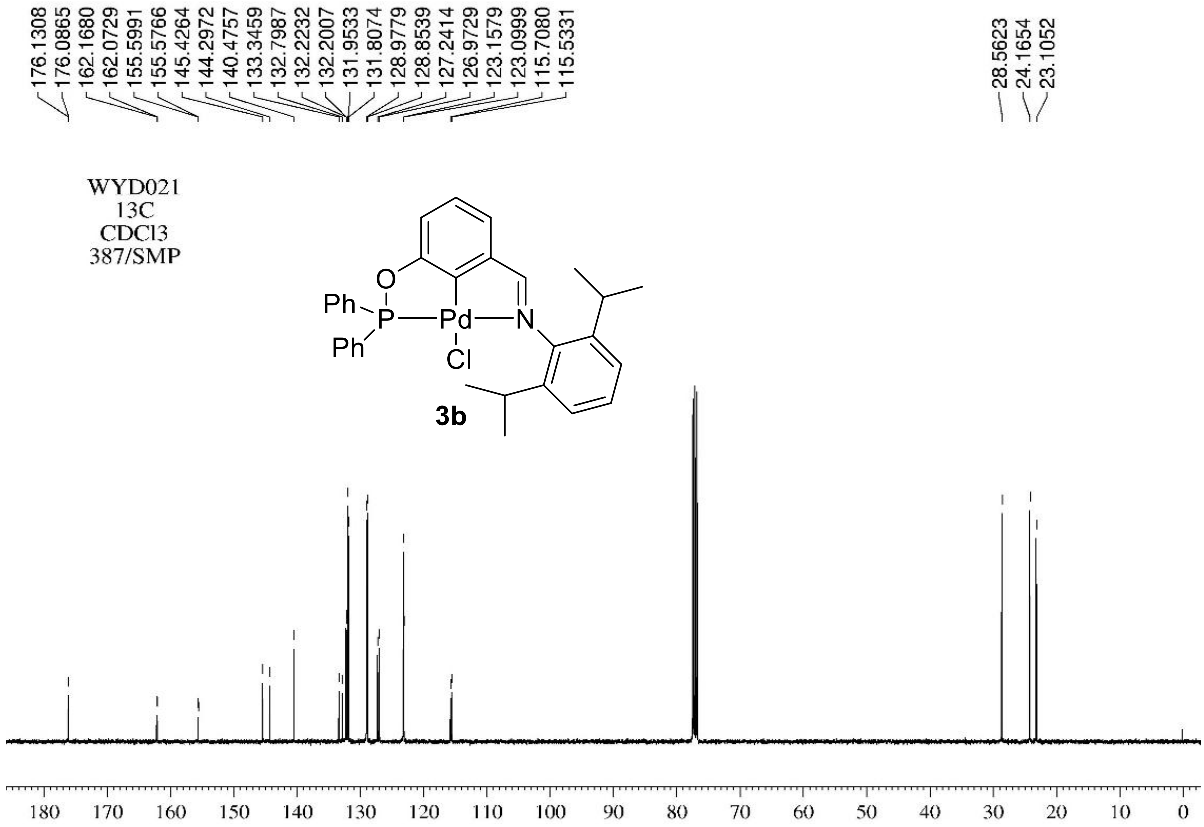

Figure S9. $^{13}C\{^1H\}$ NMR (100 MHz, $CDCl_3$) spectrum of **3b**

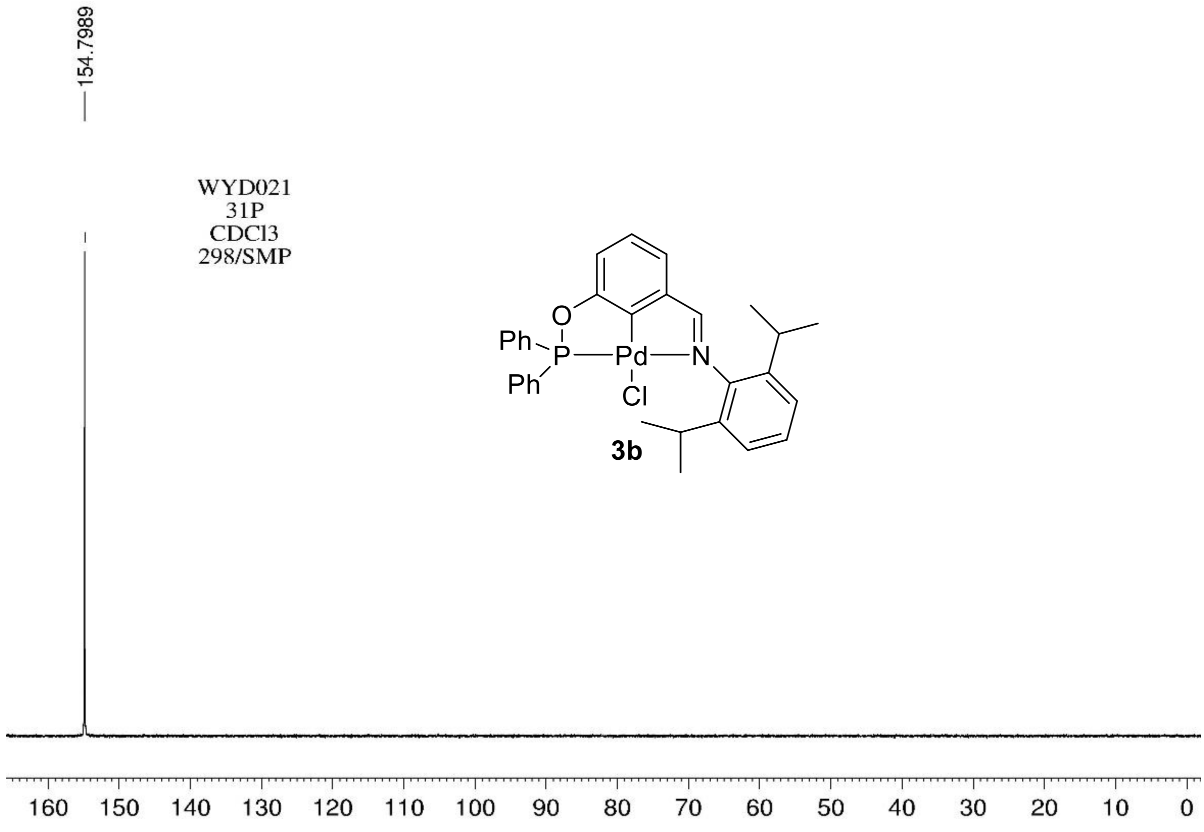

Figure S10. $^{31}P\{^1H\}$ NMR (162 MHz, $CDCl_3$) spectrum of **3b**

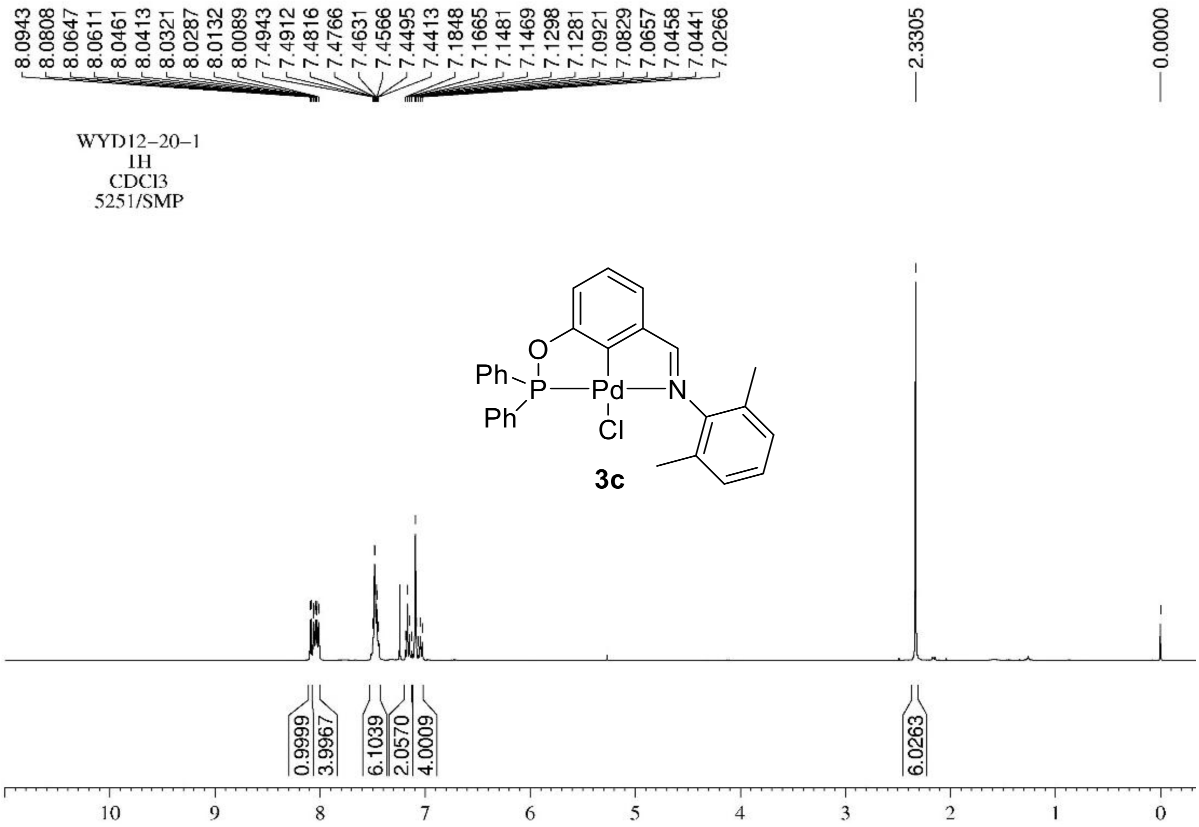

Figure S11. ^{1}H NMR (400 MHz, $CDCl_3$) spectrum of **3c**

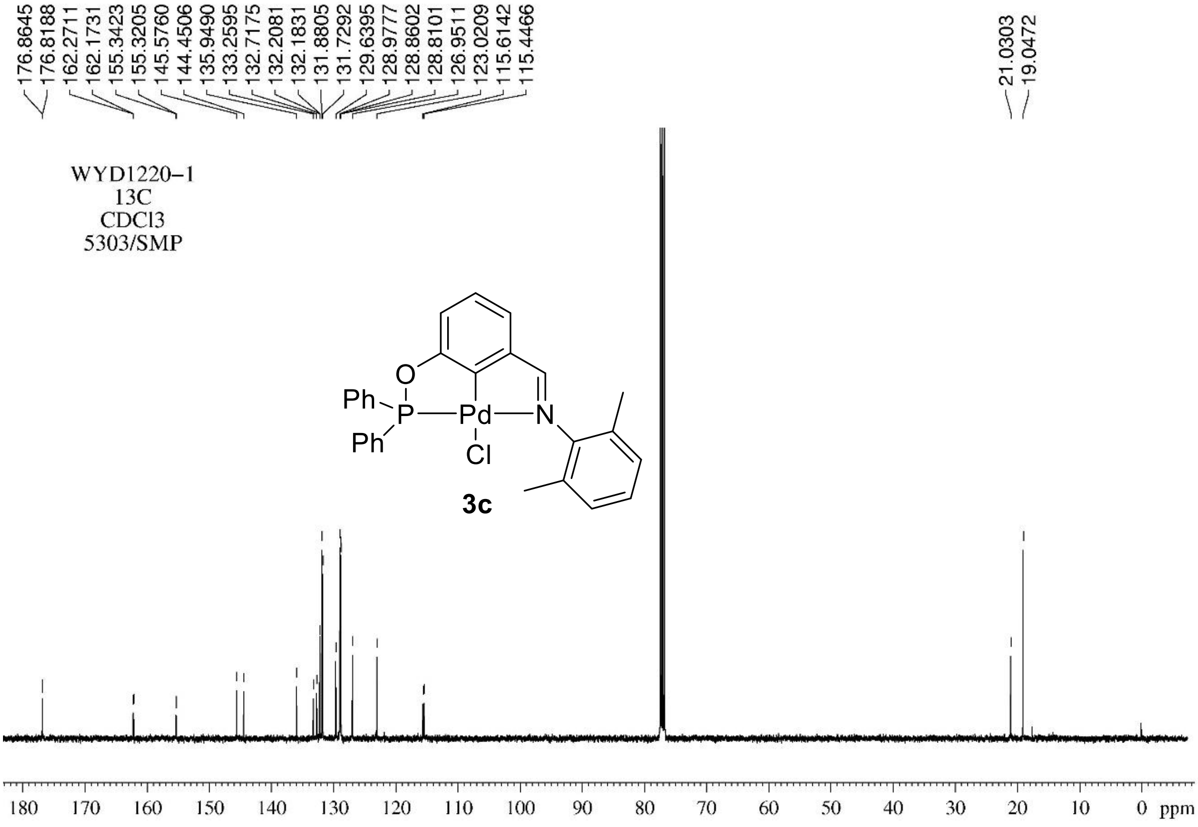

Figure S12. $^{13}C\{^{1}H\}$ NMR (100 MHz, $CDCl_3$) spectrum of **3c**

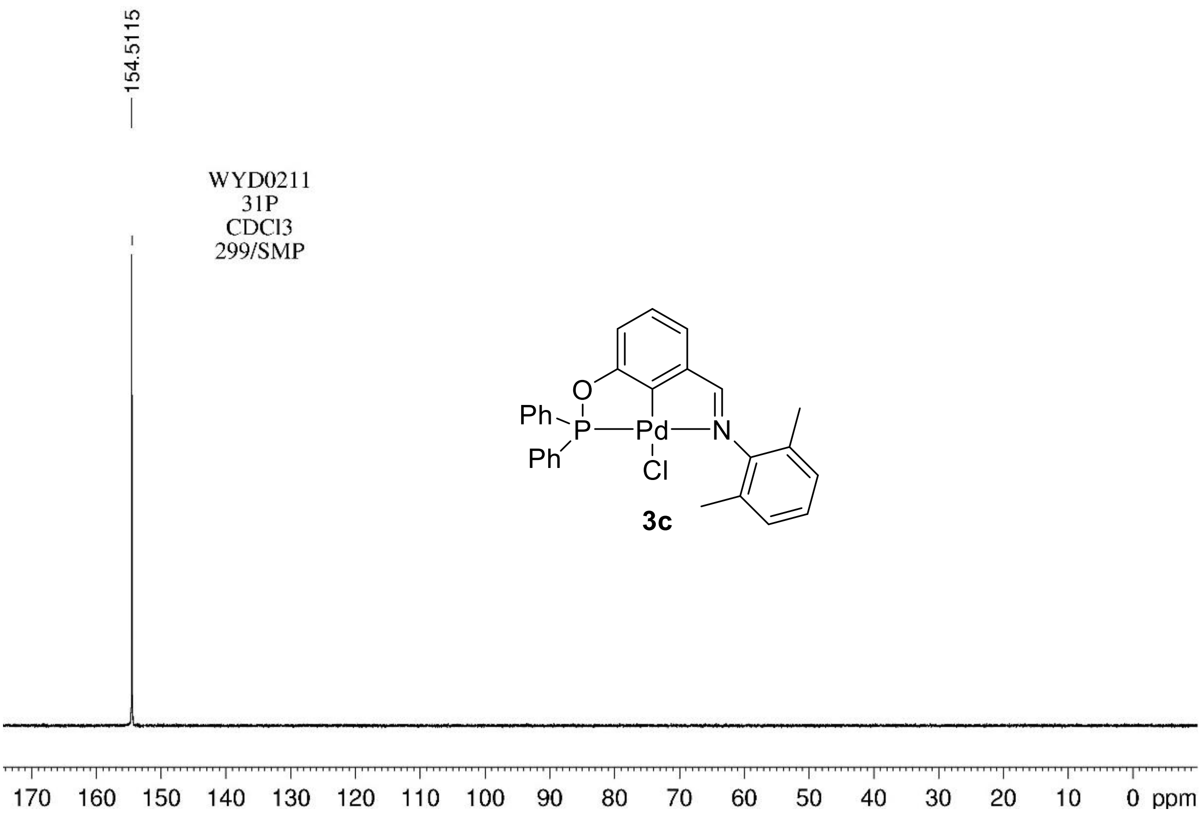

Figure S13. $^{31}P\{^{1}H\}$ NMR (162 MHz, $CDCl_3$) spectrum of **3c**

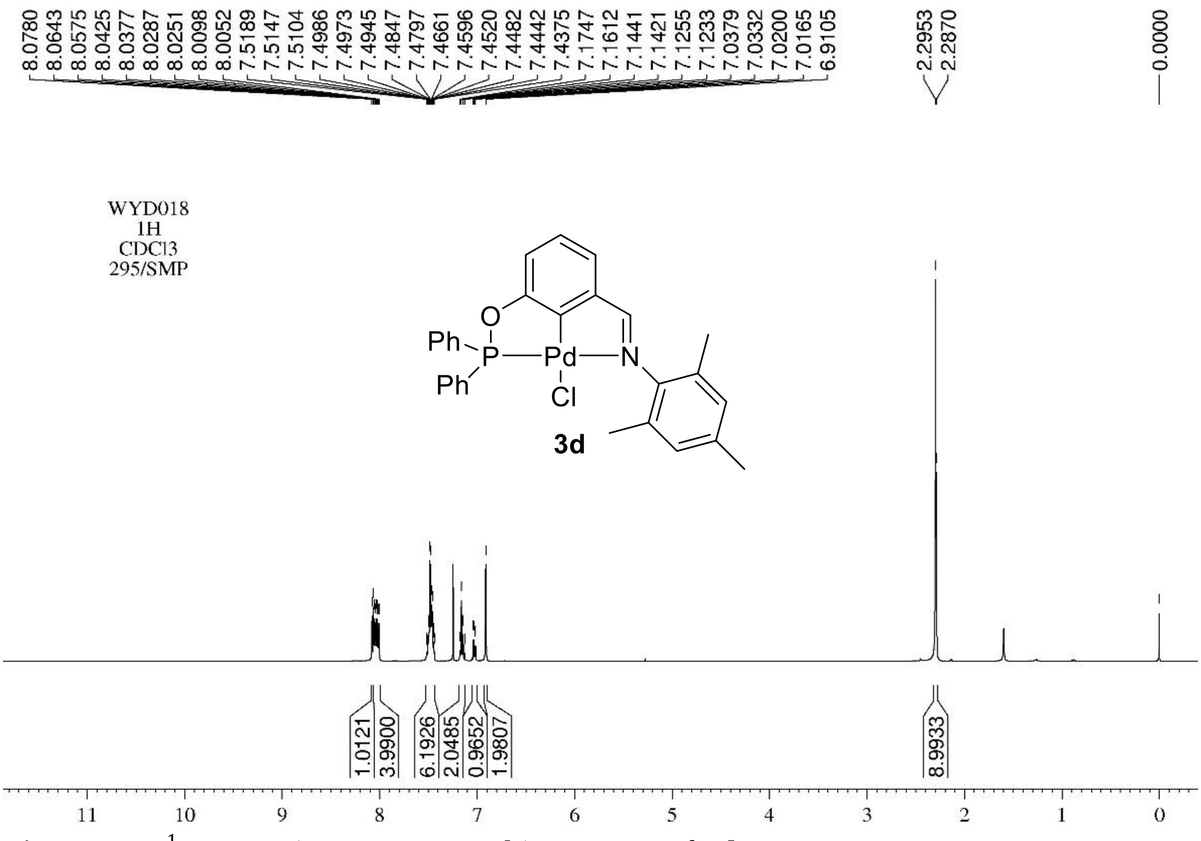

Figure S14. ^{1}H NMR (400 MHz, $CDCl_3$) spectrum of **3d**

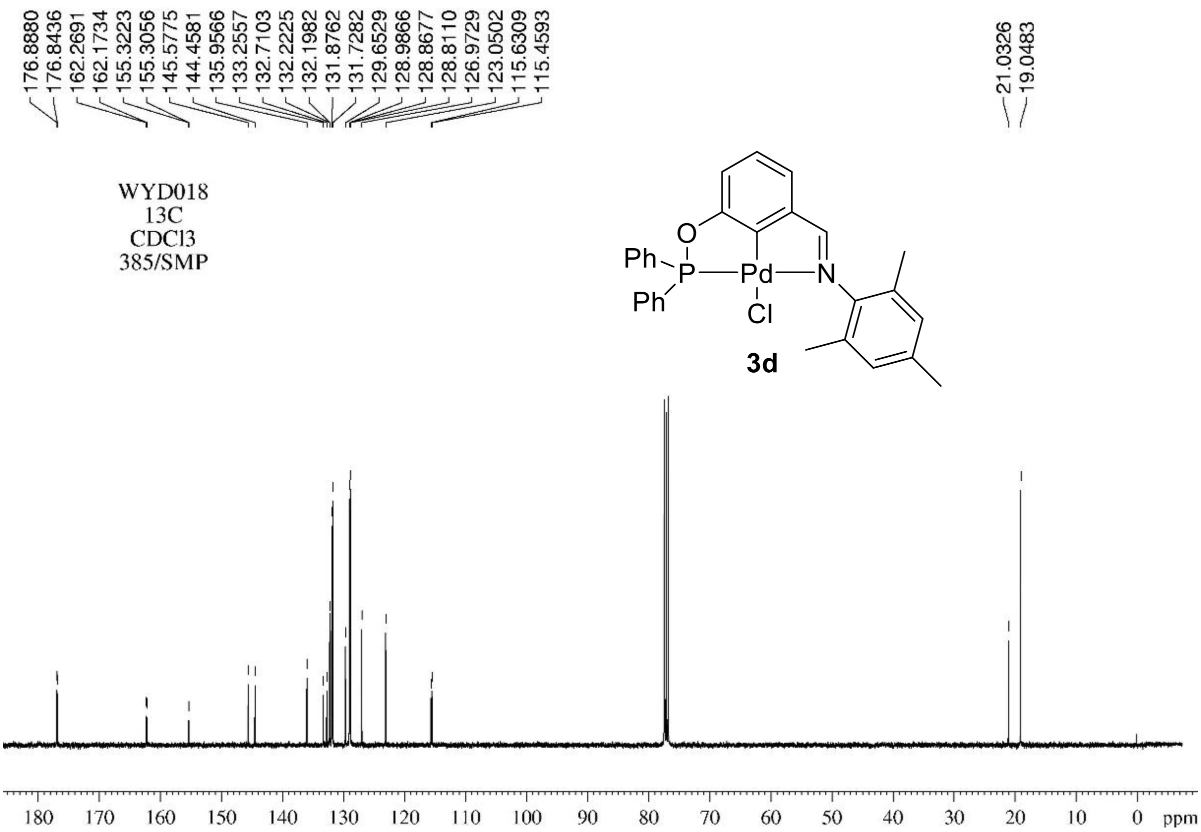

Figure S15. $^{13}C\{^{1}H\}$ NMR (100 MHz, $CDCl_3$) spectrum of **3d**

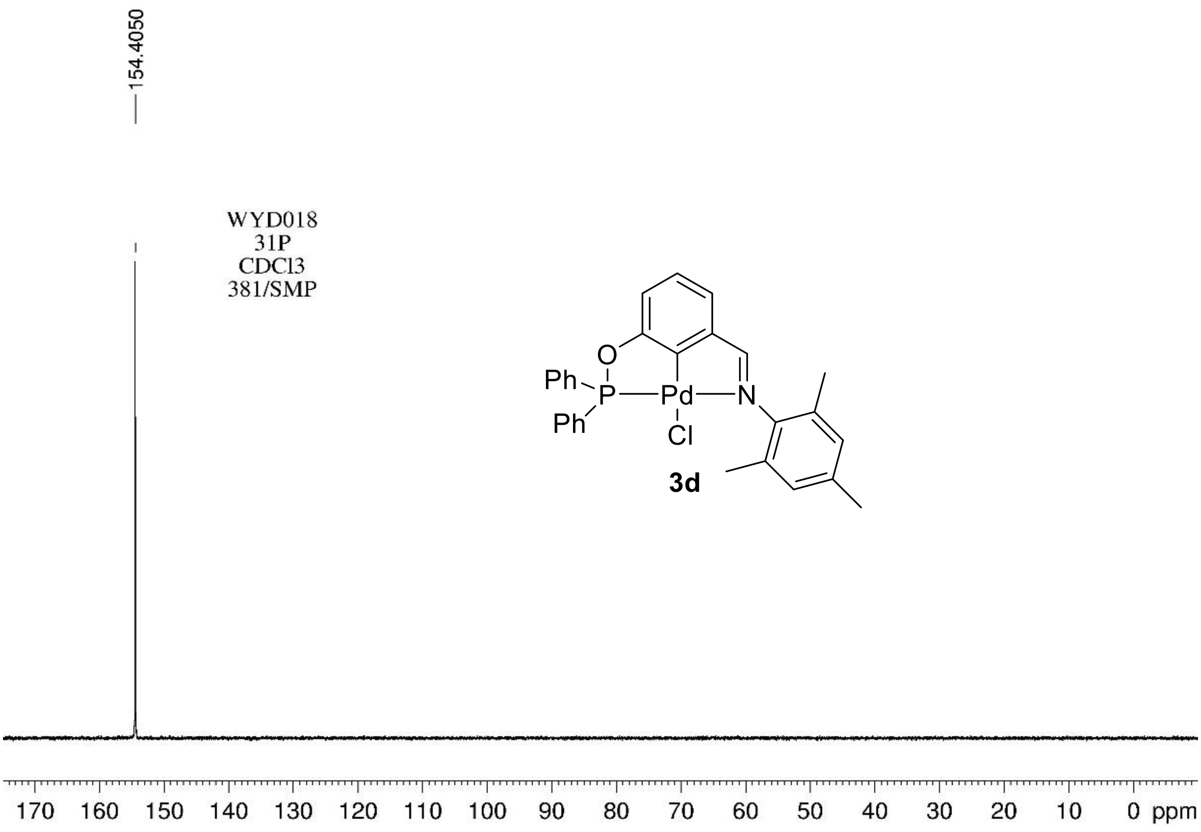

Figure S16. $^{31}P\{^{1}H\}$ NMR (162 MHz, $CDCl_3$) spectrum of **3d**

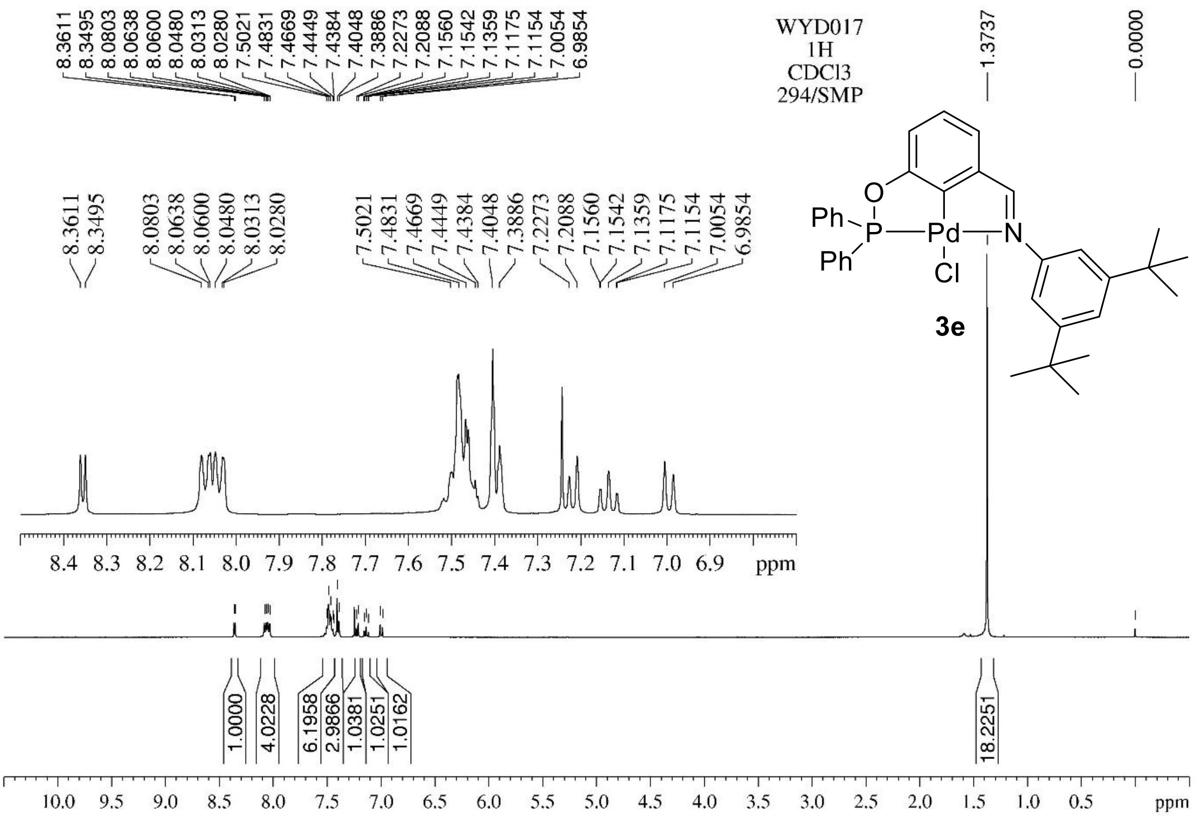

Figure S17. ^{1}H NMR (400 MHz, $CDCl_3$) spectrum of **3e**

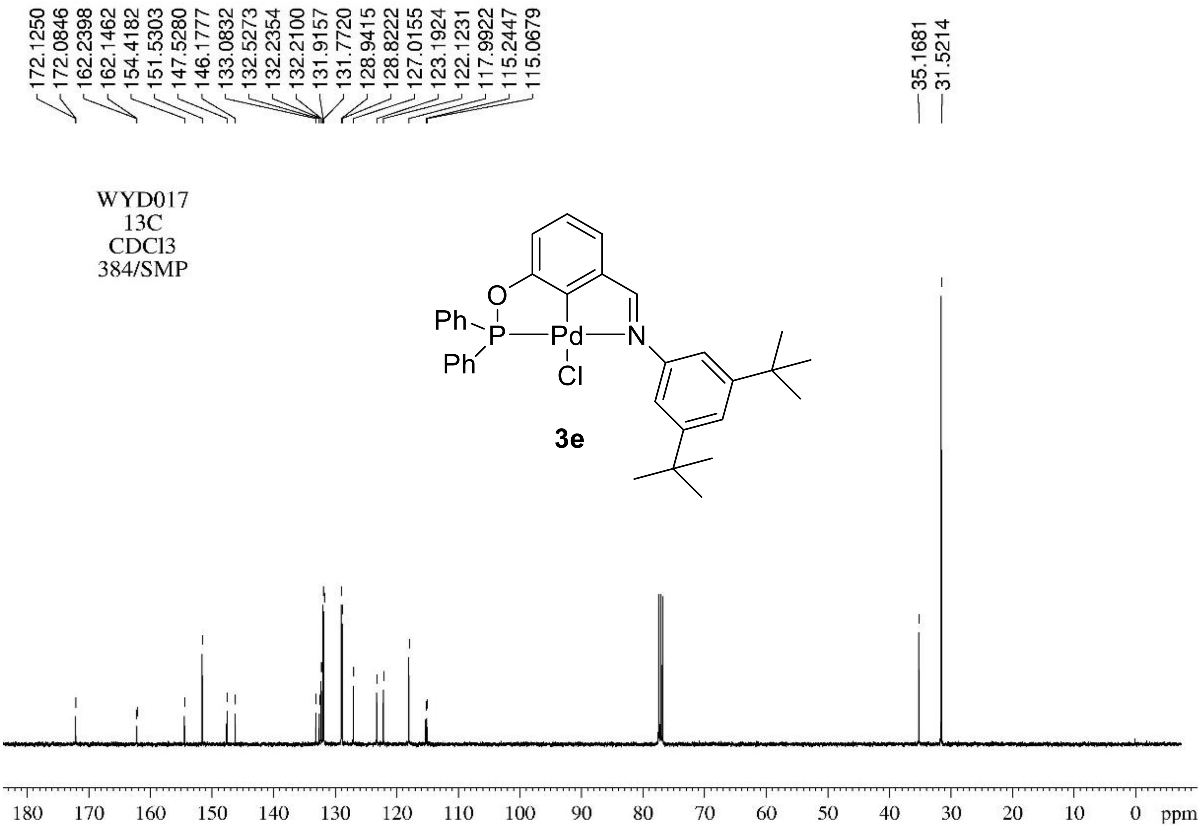

Figure S18. $^{13}C\{^{1}H\}$ NMR (100 MHz, $CDCl_3$) spectrum of **3e**

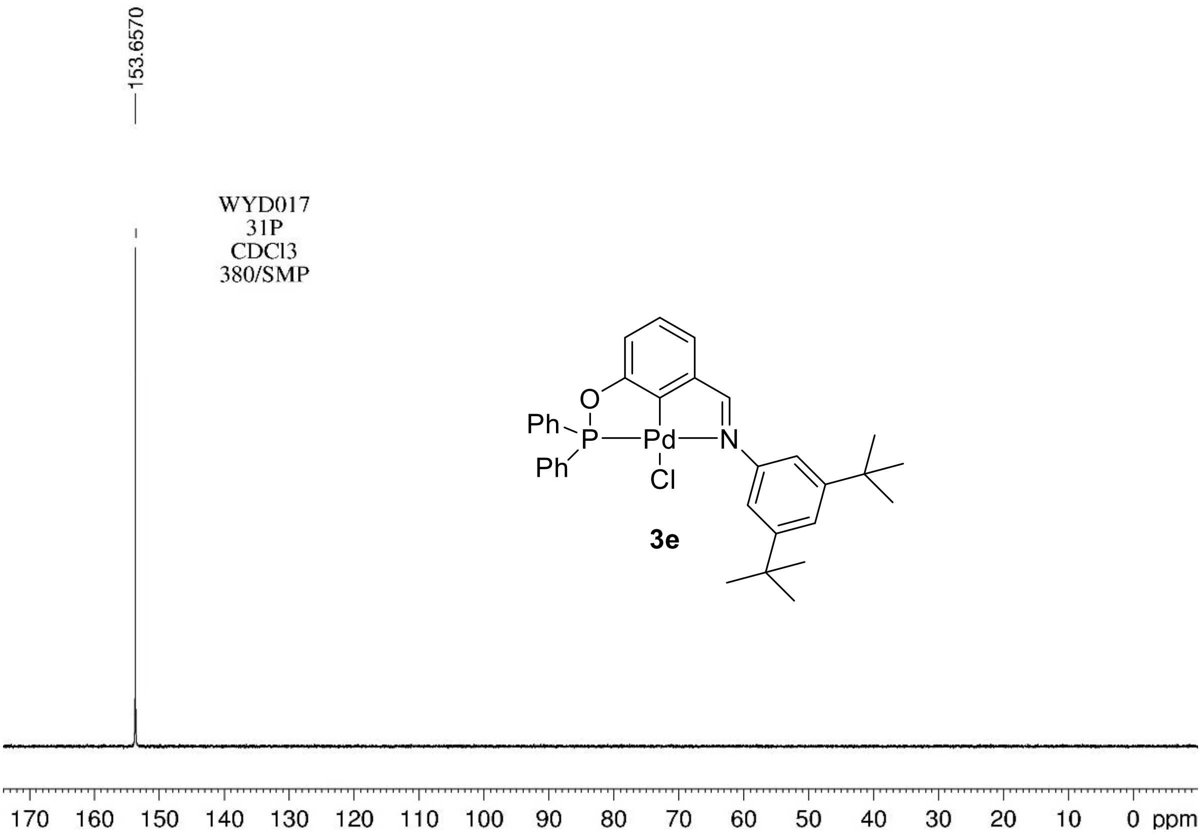

Figure S19. $^{31}P\{^{1}H\}$ NMR (162 MHz, $CDCl_3$) spectrum of **3e**

IR Spectra of norbornene homopolymers

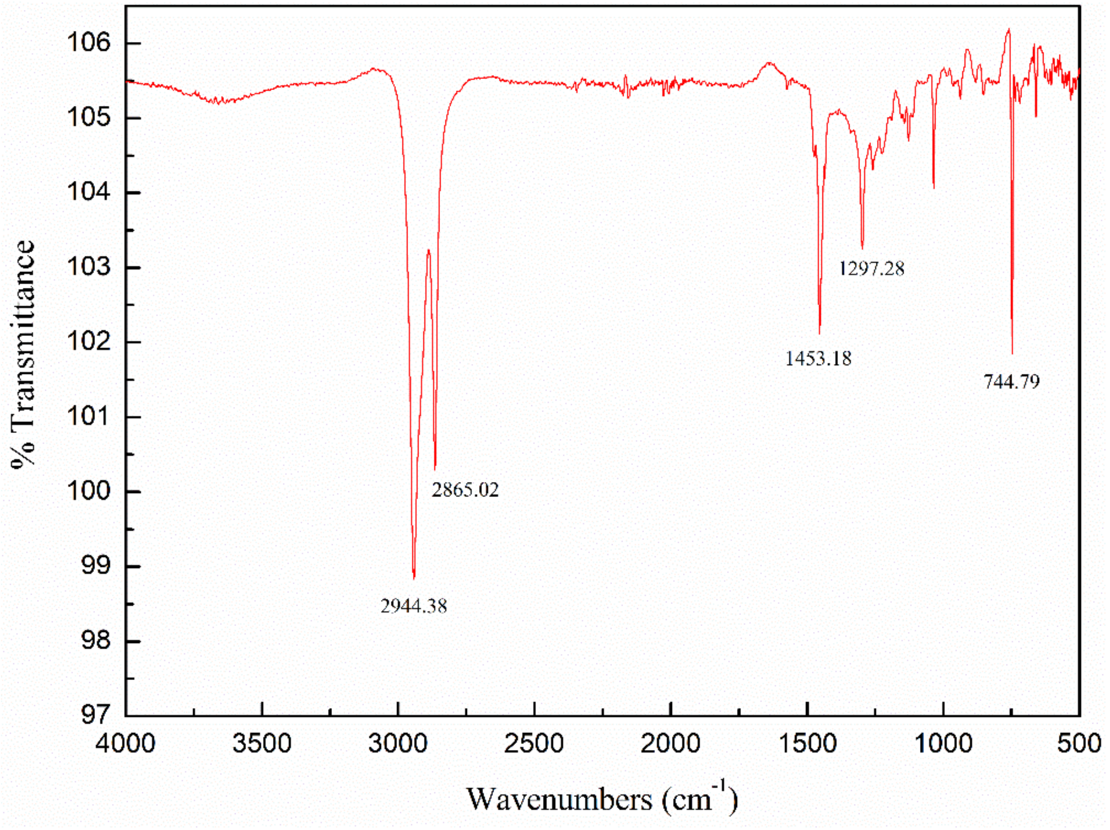

Figure S20. IR spectrum of PNB obtained by **1a**/$EtAlCl_2$ (Table 1, entry 9)

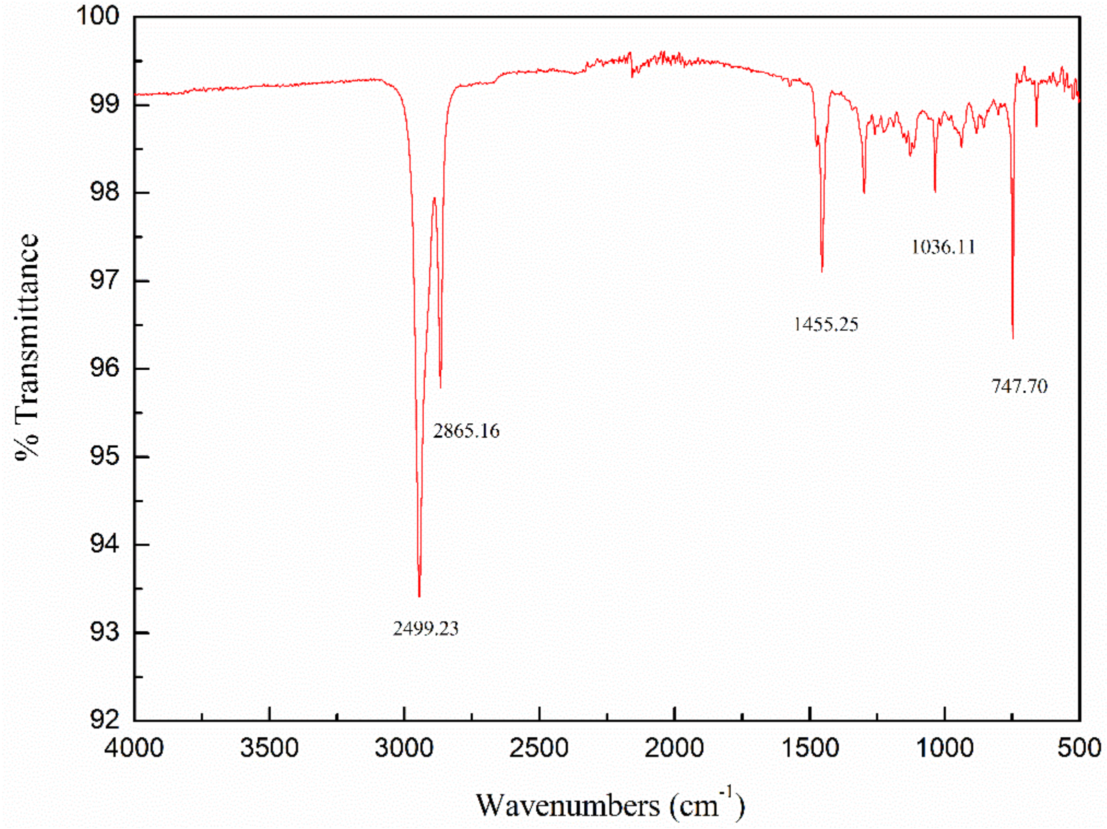

Figure S21. IR spectrum of PNB obtained by **2c**/$EtAlCl_2$ (Table 1, entry 19)

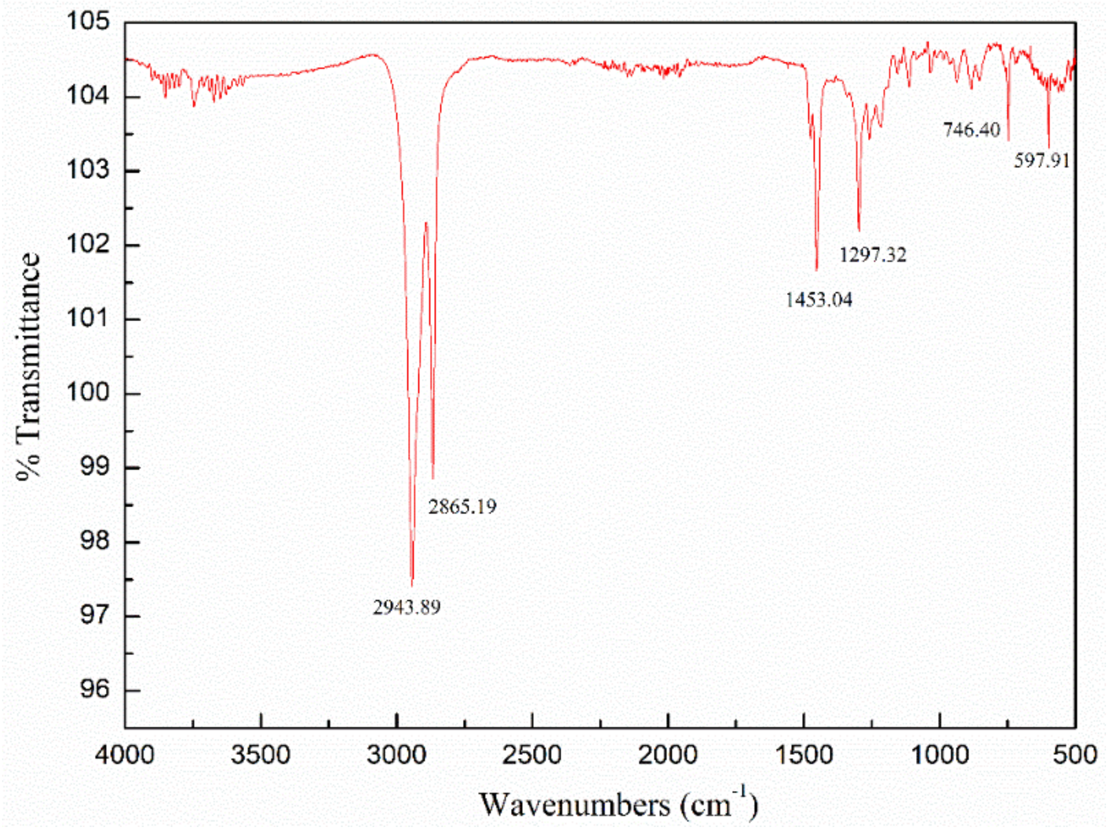

Figure S22. IR spectrum of PNB obtained by **1a**/Et_2AlCl (Table 2, entry 6)

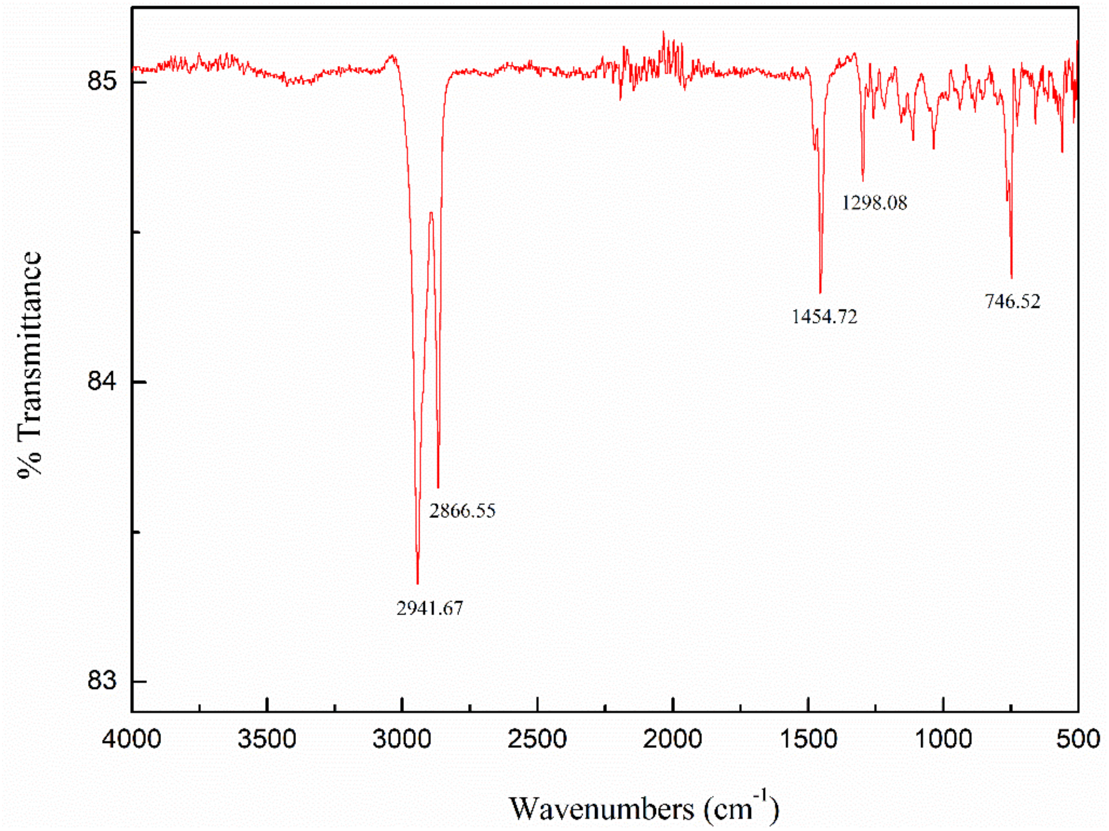

Figure S23. IR spectrum of PNB obtained by **1c**/Et_2AlCl (Table 2, entry 13)

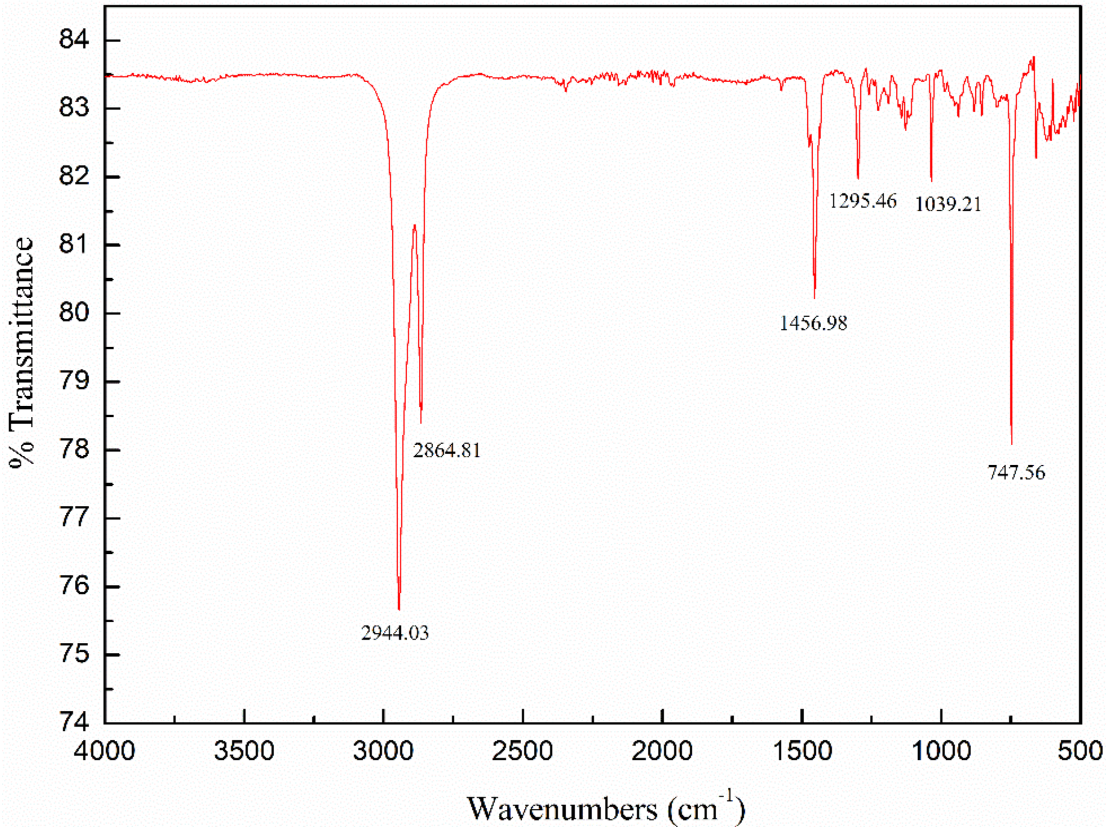

Figure S24. IR spectrum of PNB obtained by **4b**/$EtAlCl_2$ (Table 3, entry 18)

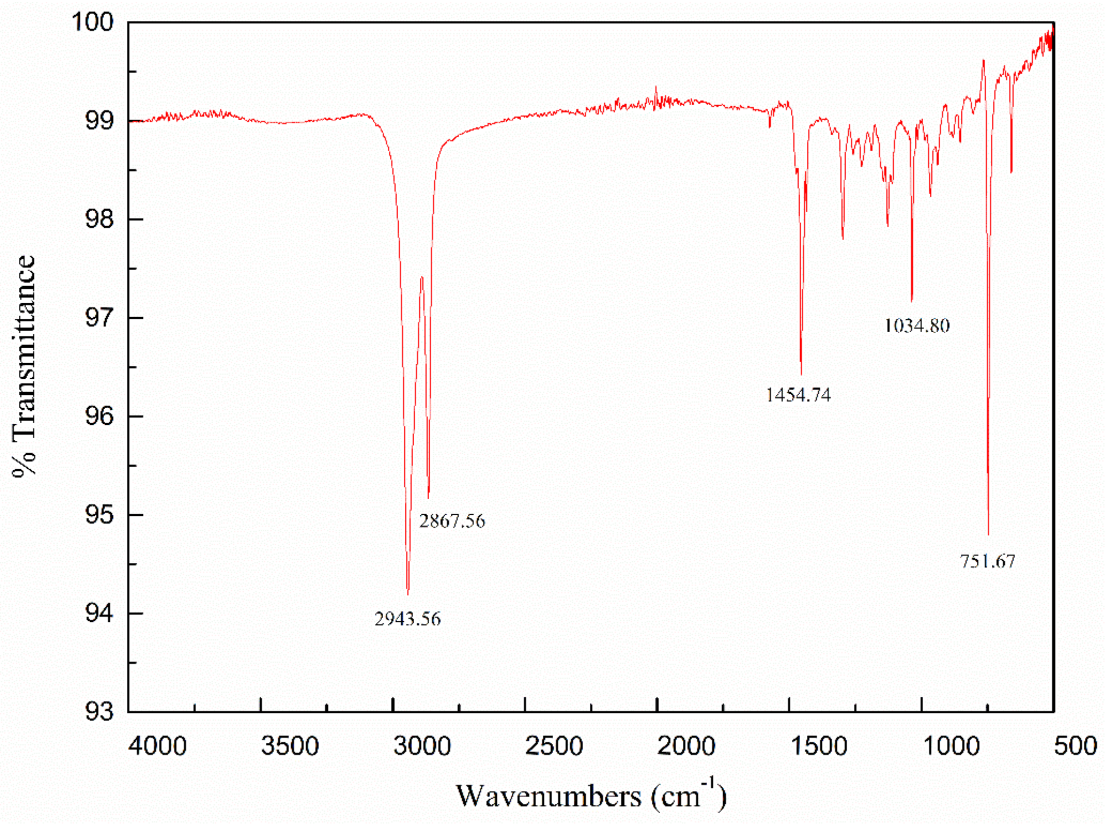

Figure S25. IR spectrum of PNB obtained by **3a**/MAO (Table 4, entry 1)

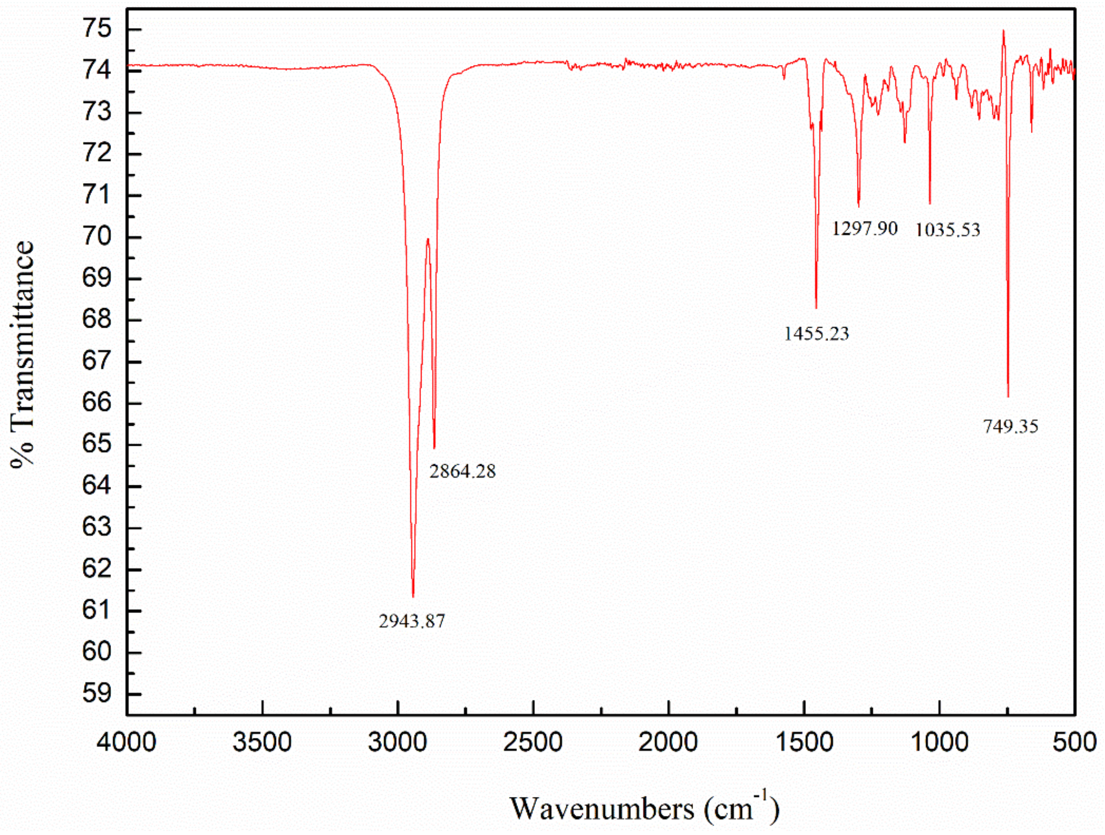

Figure S26. IR spectrum of PNB obtained by **4c**/MAO (Table 4, entry 16)

DSC data of norbornene homopolymers

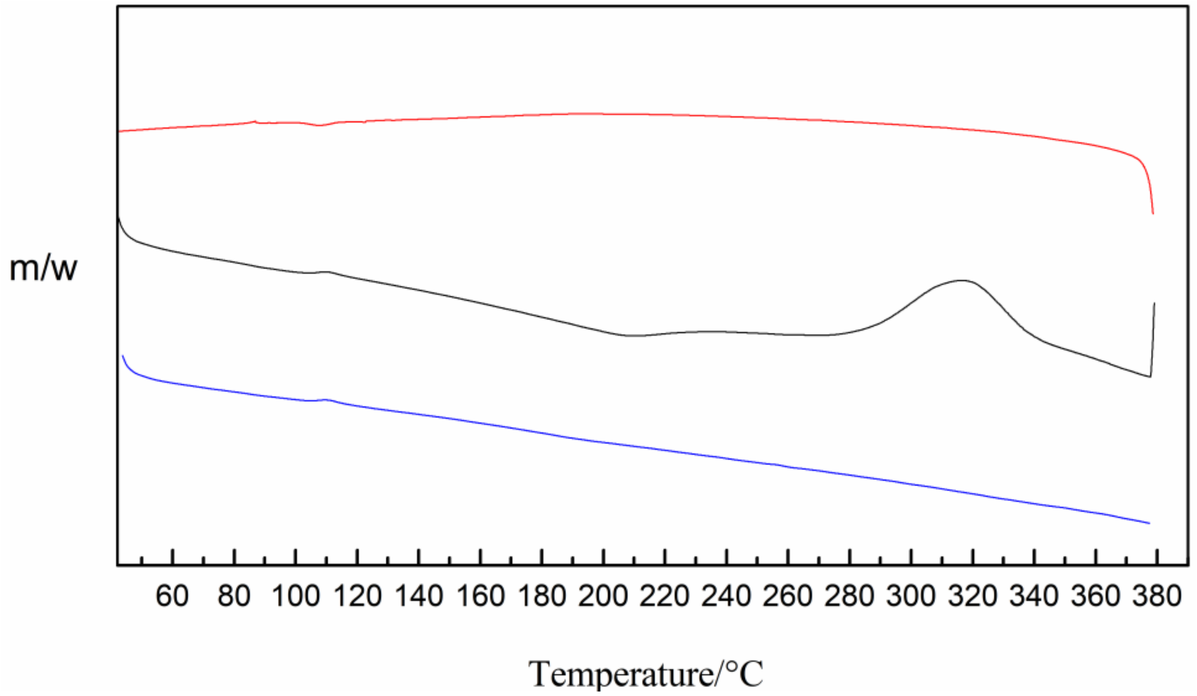

Figure S27. DSC thermograms of PNB obtained by **1a**/$EtAlCl_2$ (Table 1, entry 9)

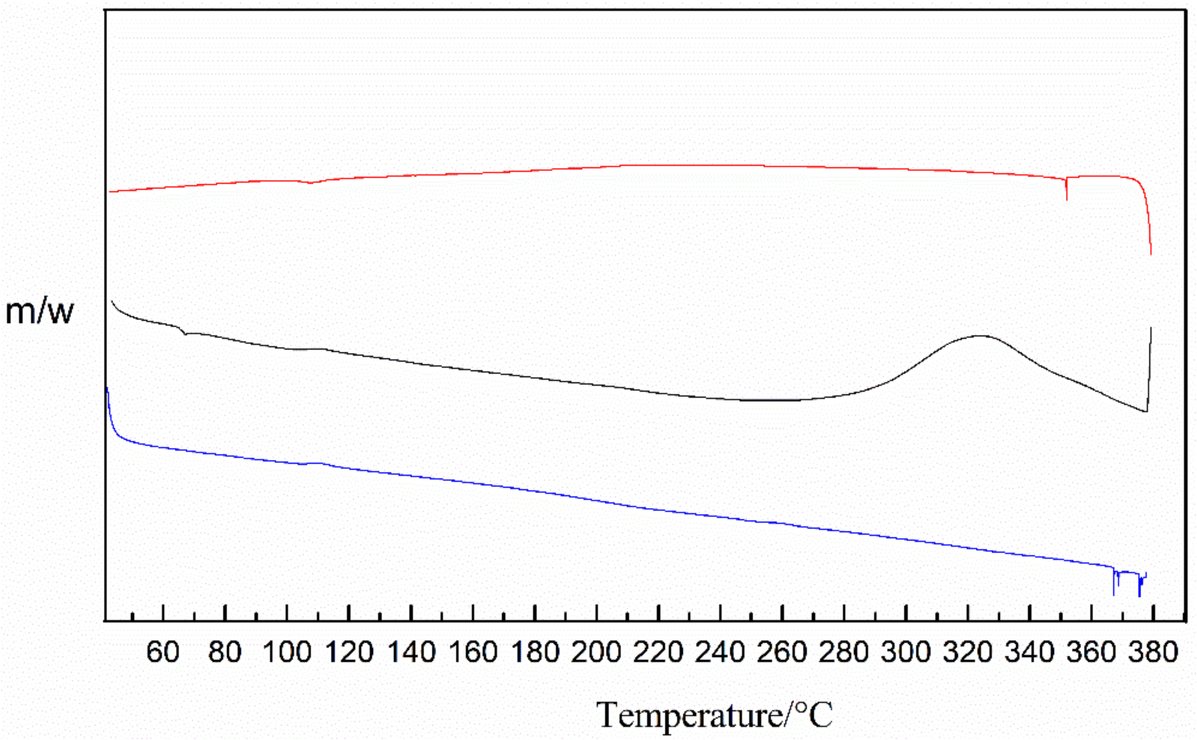

Figure S28. DSC thermograms of PNB obtained by **2c**/$EtAlCl_2$ (Table 1, entry 19)

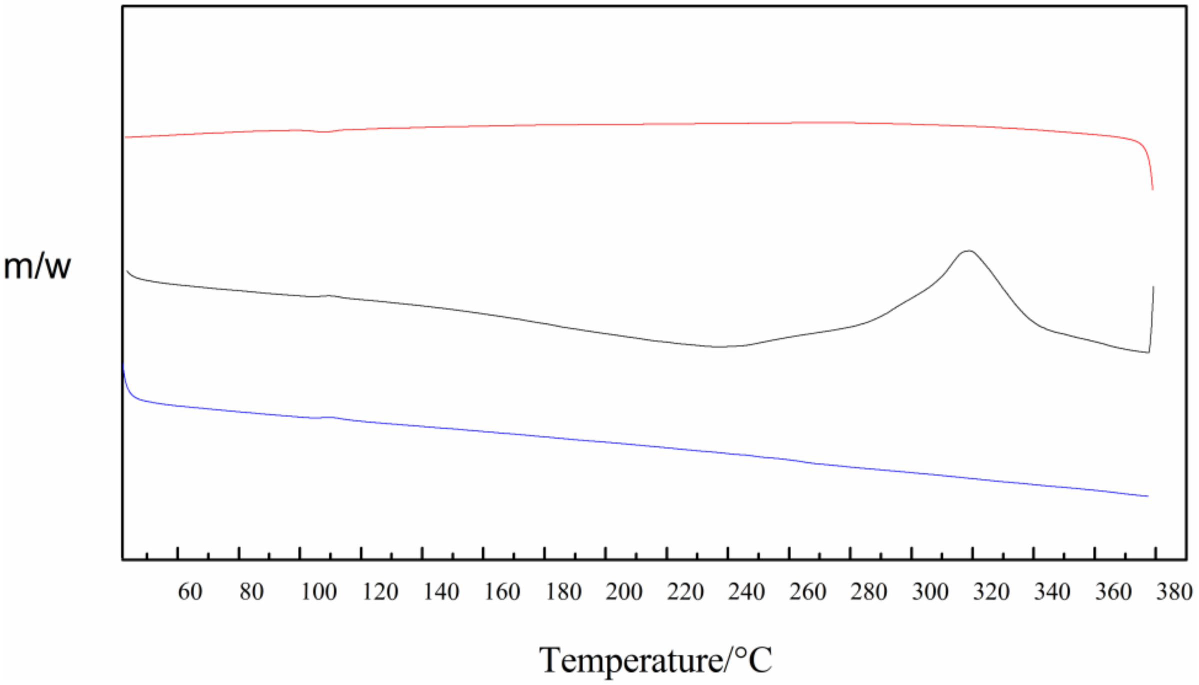

Figure S29. DSC thermograms of PNB obtained by **1a**/Et_2AlCl (Table 2, entry 6)

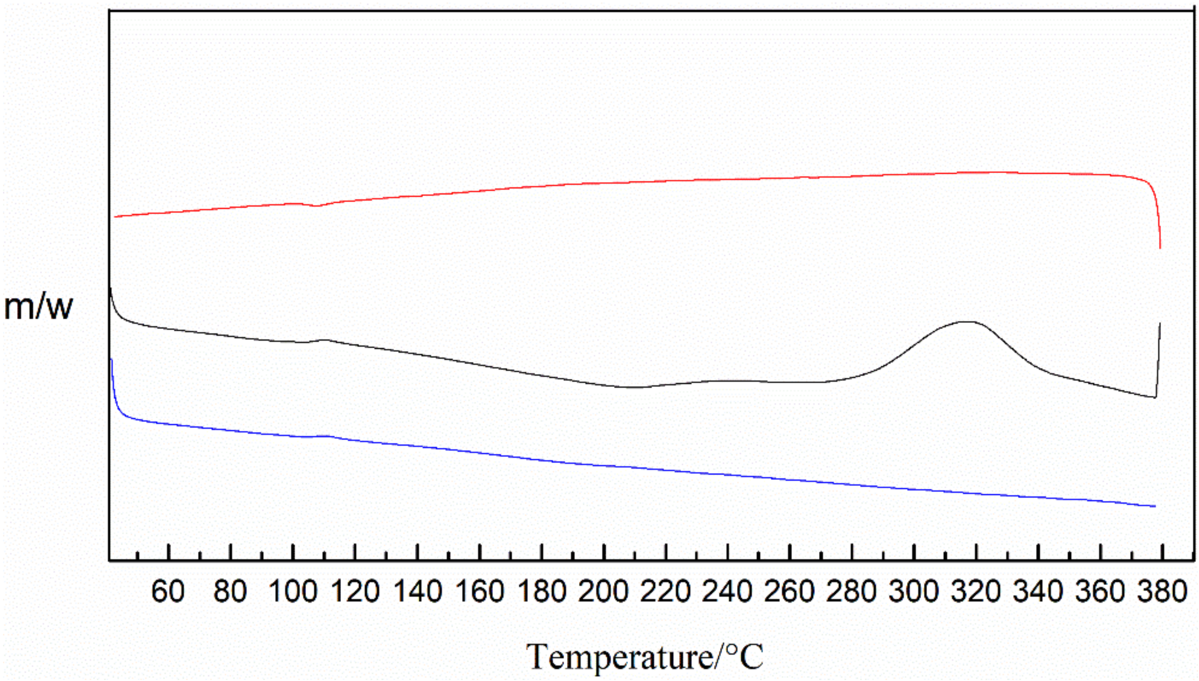

Figure S30. DSC thermograms of PNB obtained by **1c**/Et_2AlCl (Table 2, entry 13)

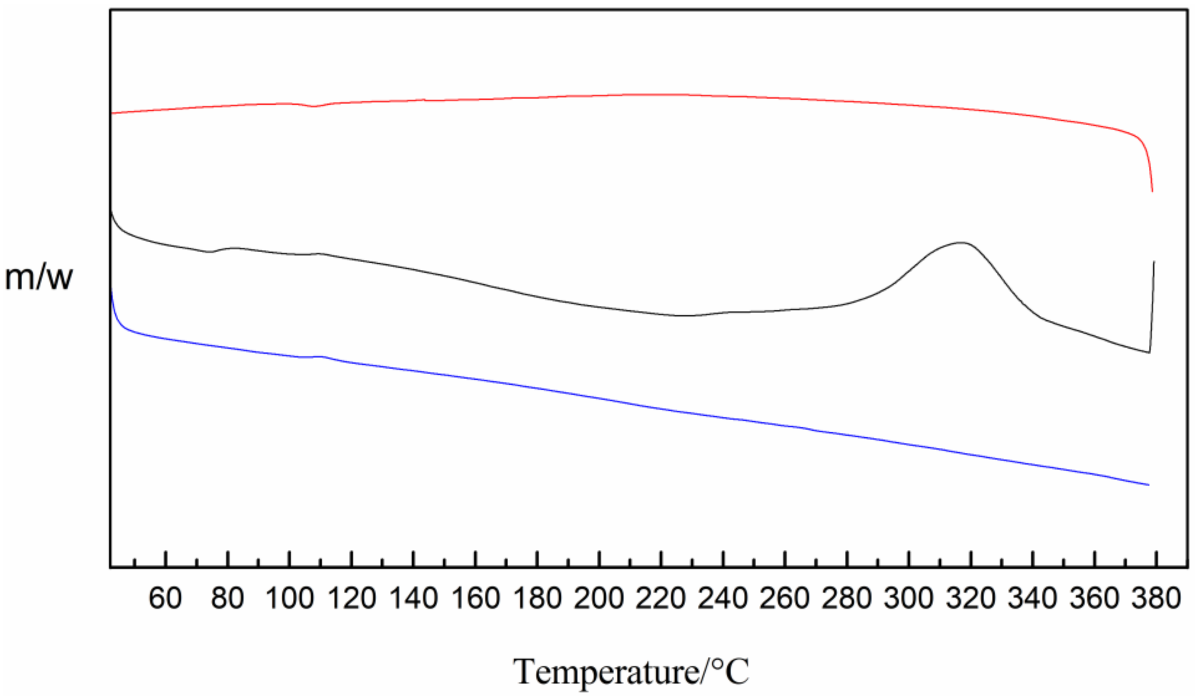

Figure S31. DSC thermograms of PNB obtained by **4c**/$EtAlCl_2$ (Table 3, entry 19)

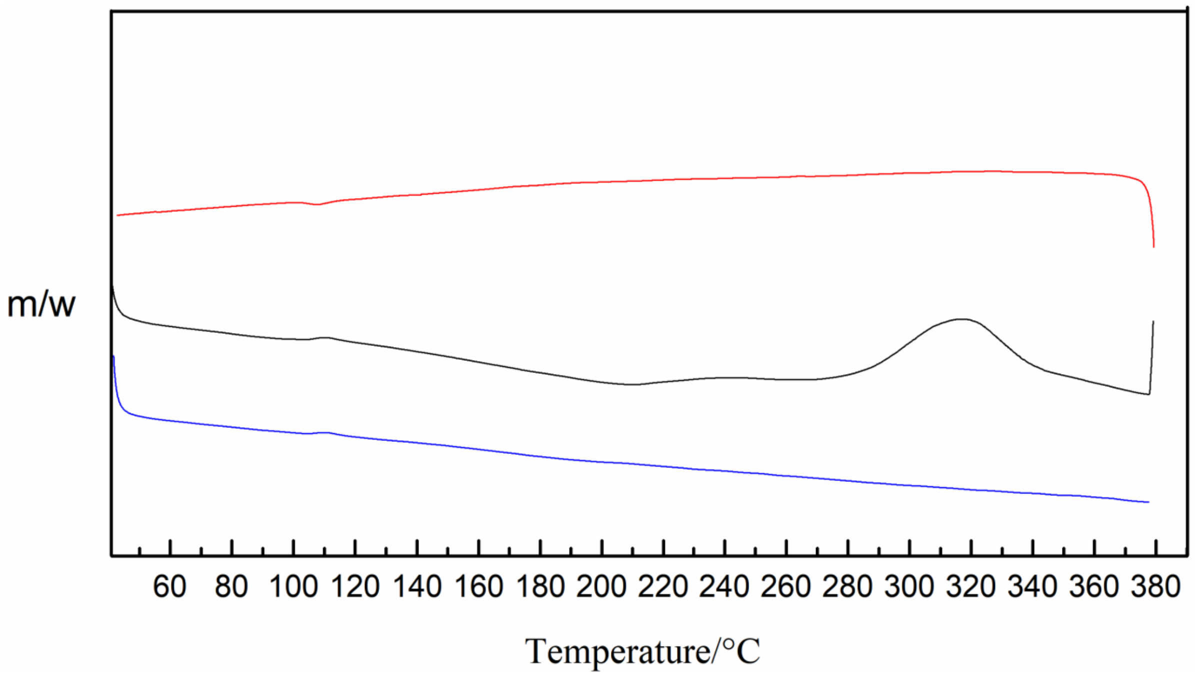

Figure S32. DSC thermograms of PNB obtained by **3a**/MAO (Table 4, entry 1)

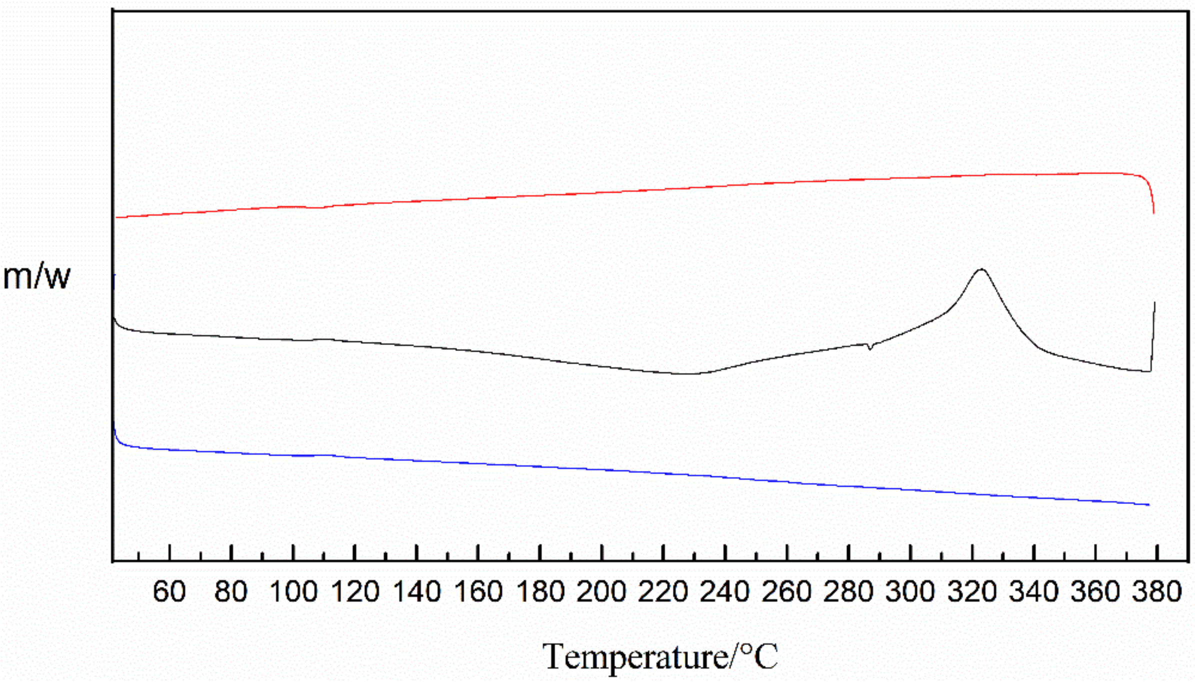

Figure S33. DSC thermograms of PNB obtained by **4c**/MAO (Table 4, entry 16)

TGA data of norbornene homopolymers

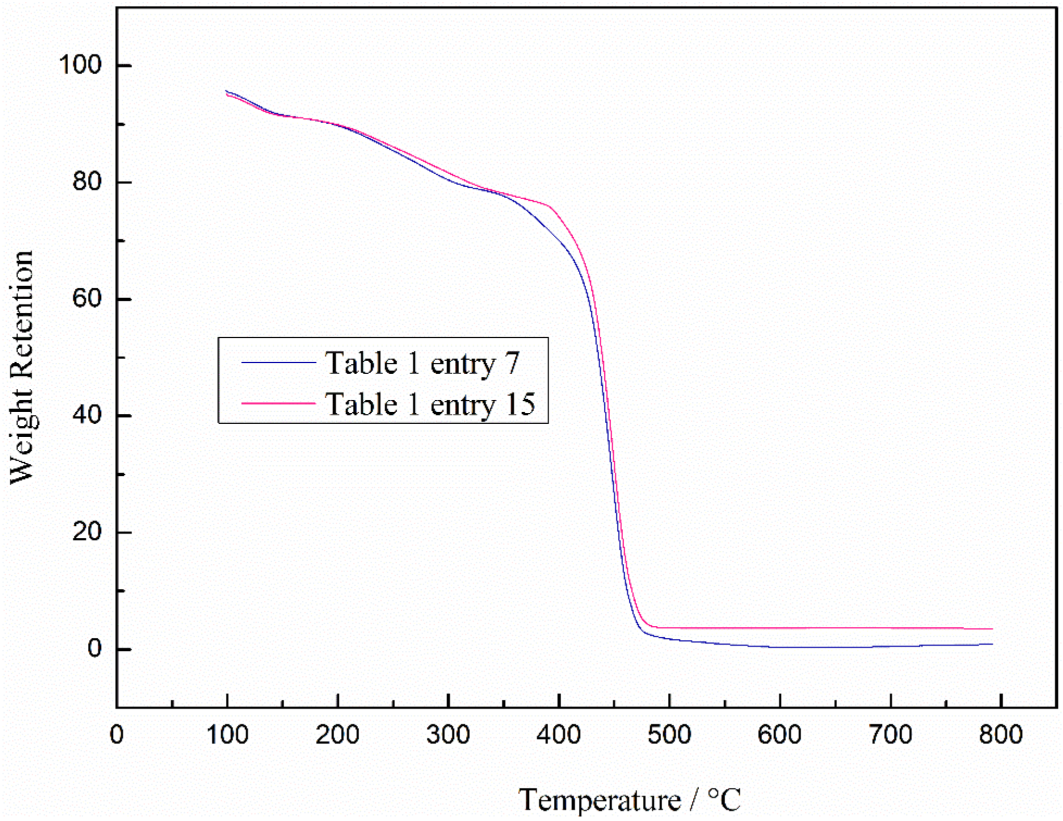

Figure S34. TGA of PNB

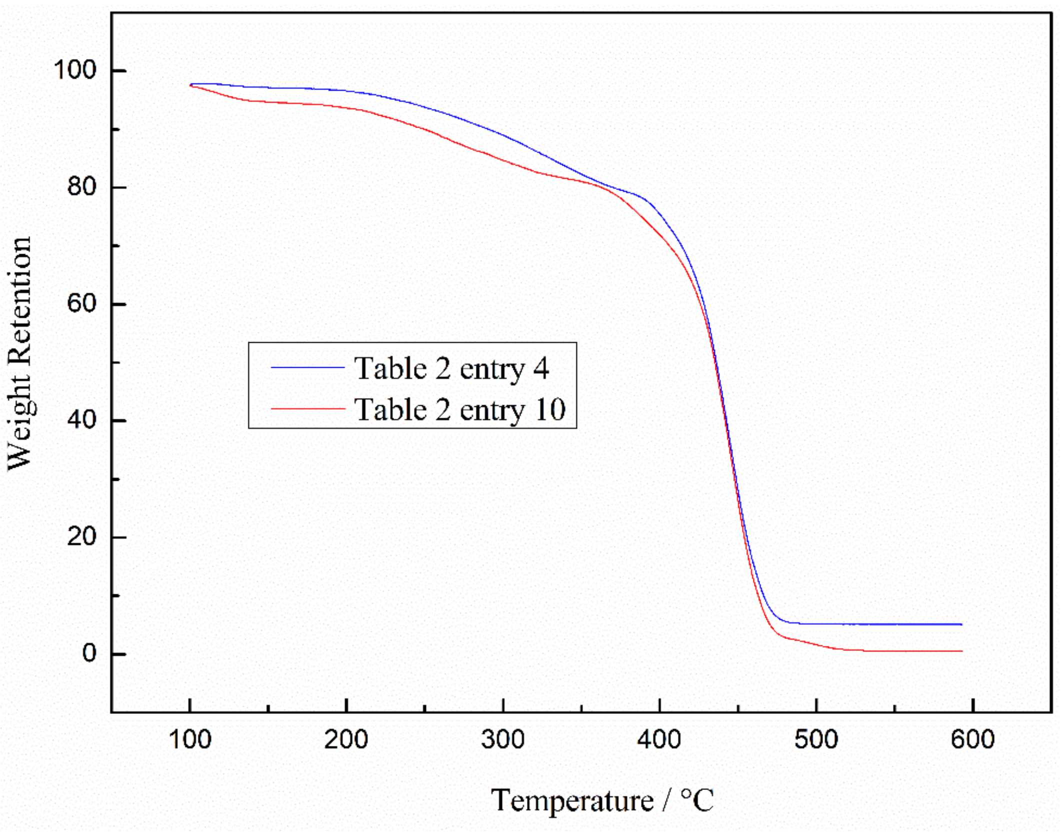

Figure S35. TGA of PNB

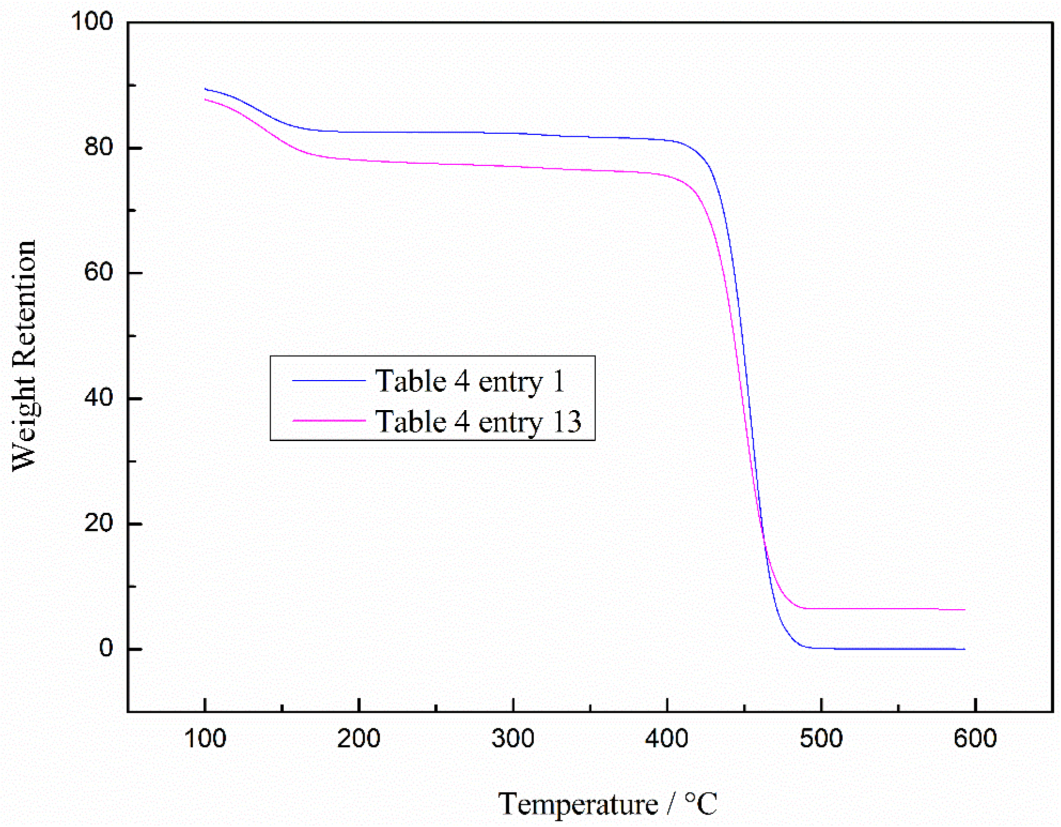

Figure S36. TGA of PNB
